# Unsterile injection equipment associated with HIV outbreak and an extremely high prevalence of HCV—A case-control investigation from Unnao, India

**DOI:** 10.1371/journal.pone.0243534

**Published:** 2020-12-04

**Authors:** Sandip Patil, Amrita Rao, Preety Pathak, Swarali Kurle, Arati Mane, Amit Nirmalkar, A. K. Singhal, Vinita Verma, Mukesh Kumar Singh, D. C. S. Reddy, Ashwini Shete, Manjula Singh, Raman Gangakhedkar, Samiran Panda

**Affiliations:** 1 Indian Council of Medical Research-National AIDS Research Institute, Pune, Maharashtra, India; 2 Uttar Pradesh State AIDS Control Society, Lucknow, Uttar Pradesh, India; 3 Community Health Centre, Department of Medical & Health, Government of Uttar Pradesh, Bangarmau, India; 4 National AIDS Control Organization, New Delhi, India; 5 Technical Resource Group, National AIDS Control Organization, New Delhi, India; 6 Indian Council of Medical Research Headquarter, New Delhi, India; University of New Mexico Health Sciences Center, UNITED STATES

## Abstract

The integrated counseling and testing center (ICTC) located in the district hospital, Unnao in the northern state of Uttar Pradesh (UP), India witnessed an increased detection of HIV among its attendees in July 2017. Subsequently, health camps were organized by the UP State AIDS Control Society in the villages and townships contributing to such detection. We conducted a case-control study to identify factors associated with this increased detection; 33 cases and 125 controls were enrolled. Cases were individuals, detected HIV sero-reactive during November 2017-April 2018 from three locations namely Premganj, Karimuddinpur and Chakmeerapur in the Bangarmau block of the district of Unnao. Controls hailed from the same geographical setting and tested HIV sero-nonreactive either in health camps or at ICTC centers from where the cases were detected. Misclassification bias was avoided by confirming HIV sero-status of both cases as well as controls prior to final analysis. Study participants were interviewed on various risk practices and invasive treatment procedures. They were also tested for HIV and other bio-markers reflecting unsafe injecting and sexual exposures such as hepatitis B surface antigen (HBsAg), anti-HCV antibody (HCV Ab), anti-herpes simplex-2 Immunoglobulin G (HSV-2 IgG) and rapid plasma regain (RPR) test for syphilis. Secondary data analysis on three time points during 2015 through 2018 revealed a rising trend of HIV among attendees of the ICTCs (ICTC-Hasanganj, ICTC-Unnao district hospital and ICTC- Nawabganj) catering to the entire district of Unnao. While there was a seven fold rise of HIV among ICTC attendees of Hasanganj (χ^2^ value for trend 23.83; p < 0.001), the rise in Unnao district hospital was twofold (χ^2^ value for trend 4.37; p < 0.05) and was tenfold at ICTC-Nawabganj (χ^2^ value for trend 5.23; p < 0.05) indicating risk of infection prevailing throughout the district. Primary data was generated through interviews and laboratory investigations as mentioned above. The median age of cases and controls was 50 year (minimum 18 –maximum 68; IQR 31–57) and 38 year (minimum 18 –maximum 78; IQR 29–50) respectively. Thirty six percent of the cases and 47% of controls were male. A significantly higher proportion of cases (85%) had HCV Ab compared to controls (56%; OR 4.4, 95% CI 1.5–12.1); none reported injection drug use. However, cases and controls did not differ significantly regarding presence of HSV-2 IgG (6% versus 8% respectively). Neither any significant difference existed between cases and controls in terms of receiving blood transfusion, undergoing invasive surgical procedures, tattooing, tonsuring of head or skin piercing. In multivariate logistic regression model, ‘unsafe injection exposure during treatment-seeking’(AOR 6.61, 95% CI 1.80–24.18) and ‘receipt of intramuscular injection in last five years’ (AOR 7.20, 95% CI 1.48–34.88) were independently associated with HIV sero-reactive status. The monophyletic clustering of HIV sequences from 14 cases (HIV-1 *pol* gene amplified) indicated a common ancestry. Availability of auto-disabled syringes and needles, empowerment of the local communities and effective regulatory practices across care settings would serve as important intervention measures in this context.

## 1. Introduction

1986 marks the year when HIV was detected first in India. A group of female sex workers (FSWs) in the southern state of Tamil Nadu was detected by HIV sero-reactive (then termed HTLV-III) [1]. The eastern metropolitan city of Kolkata in West Bengal [2, 3] also witnessed a similar phenomenon in the same year. Close to the heels of these events, a rapid rise of HIV was recorded among FSWs in Mumbai [4] in the western state of Maharashtra. Three years later, Manipur, Mizoram and Nagaland, the north-eastern states of the country bordering Myanmar, witnessed an explosive spread of HIV among people who inject drugs (PWID) [5, 6].

The aforementioned investigations helped in characterizing the concentrated nature of the HIV epidemic in most at-risk population groups (MARPs) in India and its generalized spread in a few States and also guided intervention responses. Strategic planning and operational guidelines developed by the National AIDS Control Organization (NACO) subsequently helped in the containment of the further spread of the virus [7]. However, two decades later, HIV transmission in newly identified pockets of injection drug use in the northern states of Punjab, Uttarakhand, Haryana and Uttar Pradesh (UP) as well as in the states of Odisha, Bihar, Tripura, Karnataka and Arunachal Pradesh raised an alarm [8, 9]. A striking turn of event in July 2017 added to the complexity. HIV was detected in an increasing number among attendees of the Integrated Counseling and Testing Centre (ICTC) located in the district hospital of Unnao, UP. Consequently, the medical superintendent of the hospital brought it to the notice of the district Chief Medical Officer of Health (CMOH) and sought advice. Uttar Pradesh State AIDS Control Society (UPSACS) in response to such an alert, organized health camps in the Premganj township and villages of Karimuddinpur and Chakmeerapur in Bangarmau block (blocks are the administrative subdivisions of a district in India). The first camp was held in November 2017 and an HIV test facility was offered along with health examination to the camp attendees. The decision to organize health camps in the above-specified locations was guided by the contribution of villages and township to HIV detection at the ICTC—Unnao district hospital.

The current case-control investigation was initiated against this backdrop. The overall purpose of our study was to identify factors associated with spiked detection of HIV in the above geographical settings and to suggest appropriate intervention measures.

## 2. Methodology

### 2.1 Study area

Uttar Pradesh (UP), one of the larger states of India, with 75 districts, has a population of 199,581,477 according to the census 2011 [10]. Unnao–the study district in the state—has a land area of 4,558 sq. km with a population size of 3,108,367 (population density 682 per sq. km) and literacy rates among males and females being 75% and 57% respectively. Of the total 11, 24,744 workers in the district, 40% are cultivators and 30% are agricultural laborers underlining the fact that the society is mostly agrarian [11]. Bangarmau is one of the 16 administrative blocks (subdivision) of the Unnao district catering to a population of 221563. The population of Premganj township, Karimuddinpur and Chakmeerapur villages from Bangarmau block, as per census 2011, were 1216, 728, and 630 respectively.

### 2.2 Study design and period

A case-control study was conducted from September through December 2018. To facilitate enrollment of study participants, community sensitization meetings and consultation with the local health authorities, village administration and state officials were held at different time points.

### 2.3 Ethical clearance

Approval for the current investigation was obtained from the Ethics Committee of the Indian Council of Medical Research–National AIDS Research Institute (ICMR-NARI). Written informed consent was obtained from each of the participants before interviewing them. The identity of the respondents was anonymized by ascribing unique code to each of the interview schedules and linked clinical specimens.

### 2.4 Study population

#### 2.4.1 Definition—Case

Cases were individuals detected HIV sero-reactive during the six-month-period (November 2017 to April 2018) from three study locations namely Premganj, Karimuddinpur and Chakmeerapur. Participants ≥18 year (adults) were recruited. Anti-retroviral treatment (ART) centre-Kanpur, ICTC- Hasanganj and ICTC-Unnao district hospital catered to the three study locations and records maintained by them were used to prepare the case-list. Information collected during health camps organized by UPSACS was also utilized to finalize this list.

#### 2.4.2 Definition—Control

Controls were individuals, who lived in any of the three locations as with cases and tested HIV sero non-reactive in health camps or at ICTC centers from where cases were detected during the defined period.

#### 2.4.3 Recruitment of participants

**Fig 1:**
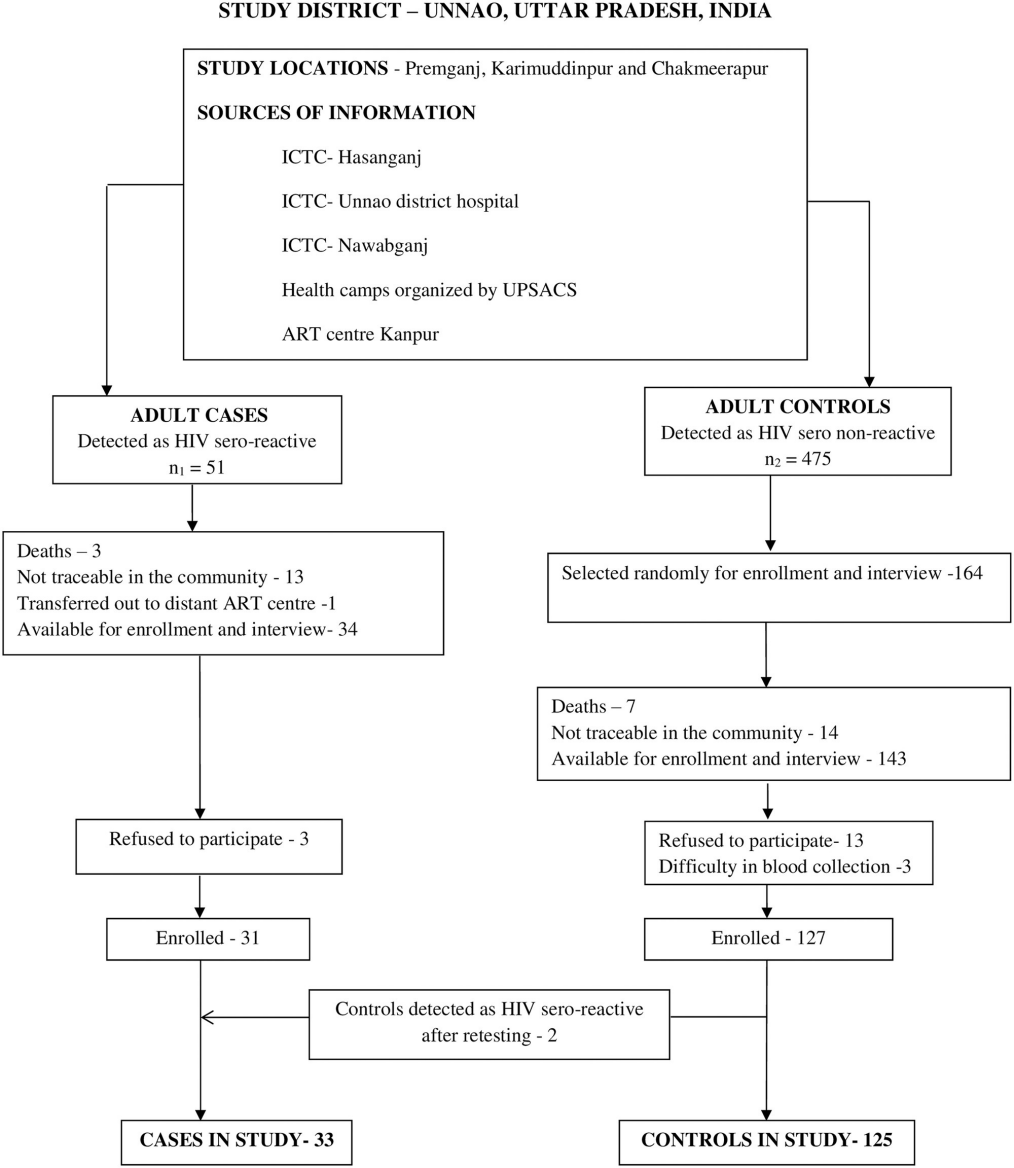
Flow chart—Recruitment of study population.

Five of the 56 cases were minors and not enrolled. Among the remaining 51 adults, 25 were males. Thirty-one cases were available for enrollment and interview. Reasons for non-participation among the rest (20/51) were deaths, non-traceability in community and refusal. Despite our plan to recruit four controls per case, refusal and difficulty in blood collection allowed the final enrollment of 127 controls. To avoid misclassification bias, we re-tested all the enrolled cases and controls for HIV. In the process, two controls were detected as HIV sero-reactive. Thus the following analysis had 33 cases and 125 controls (Fig 1).

## 3. Study tool and procedures

Being informed by two patients from the Premganj township of Bangarmau block, the medical superintendent of Unnao district hospital wrote to the CMOH that a local treatment provider had allegedly been using the same syringe and needle on different individuals for treatment. Among other things, the study questionnaire incorporated this issue as well. The district health authorities and officials from UPSACS helped in refining this study tool with the inclusion of local terminology for words such as ‘tattoo’, ‘pus’ and ‘system of medicine’. Positive HIV sero-reactive status was the dependent variable. Socio-demographic profile, substance use practices, injection exposure in therapeutic settings, sexual practices, symptoms of sexually transmitted diseases (STDs), blood transfusion, surgical interventions, dental procedure, barber service utilization, tattooing, tonsuring, skin piercing practices and illness histories were inquired upon as independent variables.

Male and female interviewers, identified from a nearby locality, were trained on the structure and content of the finalized interview schedule through mock and supervised interview sessions. Male and female participants were interviewed by male and female interviewers respectively.

### 3.1 Serologic investigations

Seven milliliters of blood was collected from each study participant in an Ethylene Diamine Tetra Acetic acid (EDTA) vacutainer tube. Plasma separation was carried out by trained project staff at ART-centre, Kanpur and Community Health Centre (CHC) Bangarmau, which served as interview sites as well. Specimens were stored at -18°C till transportation in dry ice to ICMR-NARI, Pune.

HIV antibody was detected by using a rapid diagnostic method (Meriscreen HIV1-2 and Signal HIV) as well as the Enzyme-Linked Immunosorbent Assay (ELISA) (Microlisa HIV). Antibody against hepatitis C virus (anti-HCV IgG) was tested using Ortho HCV 3.0 ELISA (Ortho Clinical Diagnostics, USA). All HCV sero-reactive specimens were further tested by the Xpert HCV viral load assay (Cepheid, USA). Hepatitis B surface antigen was detected using Murex HBsAg Version 3.0 ELISA (Diasorin, Italy). Rapid Plasma Reagin antibody kit (Arkray Healthcare Pvt Ltd, India) and HSV-2 IgG ELISA test system (DIAPRO, Italy) were used for detecting syphilis and exposure to herpes virus type 2 infection respectively.

### 3.2 HIV-1 pol gene sequencing

Viral RNA extraction was done from the plasma using the NucliSens EasyMag total nucleic acid extraction system (Biomerieux). The extracted RNA was amplified for complete HIV-1 protease and partial reverse transcriptase region as described previously [12] and sequenced using Genetic Analyzer (Applied Biosystems 3130XL, Thermo Fisher Scientific). Sequence assembly and base calling was performed using SeqScape v2.6 software (Thermo Fisher Scientific). Sequence FASTA files generated were employed for further analysis.

### 3.3 Data analyses

Analysis of the trend of HIV case detection in all three ICTCs (Hasanganj, Unnao district hospital, and Nawabganj) and examination of primary data generated through one-on-one interviews and blood tests were two principal investigation approaches. Paper-based forms were used to capture interview responses, which were checked for their quality daily and computerized following necessary corrections. Distributions of cases and controls across various exposures were compared. Association between exposure variables and HIV sero-reactive status were examined through the Mantel-Haenszel estimate of the odds ratio. Biologically plausible variables and variables bearing significant association (p < 0.1) with the study outcome were entered into a logistic regression model using STATA (version 10.0/10.1; StataCorp, College Station, TX).

## 4. Results

### 4.1 Secondary data analysis

ICTC data generated by UPSACS during 2015–2018 were used. Significantly rising trends of positive HIV sero-reactive status among attendees of all three ICTC centers (Hasanganj, Unnao district hospital and Nawabganj) were evident (Table 1).

**Table 1:** HIV seroreactivity among ICTC attendees, Unnao district.

ICTC centers, Unnao district	HIV test	2015–16	2016–17	2017–18	Chi-square value (trend)	p-value
CHC Hasanganj	Positive	3	10	42	23.83	< 0.001
	Negative	1287	1501	2065		
District hospital, Unnao	Positive	53	47	82	4.37	0.03
	Negative	6528	6033	7058		
CHC Nawabganj	Positive	1	4	6	5.23	0.02
	Negative	1431	1515	948		

### 4.2 Participants’ profile

The median age of the cases was 50 year and mean 46 year (minimum 18; maximum 68, IQR 31–57 year), and that of the controls was 38 year and 40 year respectively (minimum 18; maximum 78; IQR 29–50 year). About a third of the cases (12/33) and 47% of controls (59/125) were males. Nearly half of the cases and controls never attended a school.

None of the cases and controls during study participation reported ‘being away from home for work’. About a fifth of the cases (6/33) reported ever staying away from home for 3 months or more. Controls (26/125; 21%) did not differ significantly with cases in this regard. While more than half of the cases reported being unemployed, about a third of the controls reported so (OR 2.38; 95% CI 0.98–5.79; p = 0.055, Table 2). The majority of the males in cases made a living either through farming or as a daily wage laborer (10/12; 83%). Similar was the profile of occupational engagement among controls (39/59; 66%). Most of the female participants among cases (17/21; 81%) were housewives whereas a lesser proportion in controls reported so (42/64; 66%).

**Table 2:** Socio-demographic profile, sexual practices* and injection exposure.

Practices	Cases	Controls	OR^a^ (95% CI^b^)	p-value
	n (%)	n (%)		
**Age**				
> 37 year	23 (69.7)	69 (55.2)	1.86 (0.82–4.25)	0.137
≤ 37 year	10 (30.3)	56 (44.8)	Reference	
**Residence**				
Premganj	18 (54.5)	62 (49.6)	0.48 (0.10–2.22)	0.351
Chakmeerapur	12 (36.4)	58 (46.4)	0.34 (0.07–1.64)	0.181
Karimuddinpur	3 (9.1)	5 (4)	Reference	
**Occupation**				
Unemployed	19 (57.58)	46 (36.80)	2.38 (0.98–5.79)	0.055
Famer	5 (15.15)	27 (21.60)	1.06 (0.33–3.50)	0.911
Non-agricultural	9 (27.27)	52 (41.60)	Reference	
**Ever had sex with a female casual partner as reported by male participants**				
Yes	2 (20.0)	4 (6.9)	3.38 (0.53–21.52)	0.19
No	8 (80.0)	54 (93.1)	Reference	
**Condom use during last sex**				
No	24 (82.8)	91 (80.5)	1.16 (0.39–3.38)	0.785
Yes	05 (17.2)	22 (19.5)	Reference	
**Received intramuscular injection in last five years**				
Yes	31 (94.0)	79 (63.2)	9.02 (2.06–39.46)	0.003
No	02 (6.0)	46 (36.8)	Reference	
**Syringe & needle during intramuscular injection in the last five years**				
Didn’t notice if the syringe & needle were new	6 (18.18)	11 (8.8)	3.67 (1.18–11.32)	0.024
Injected by used syringe & needle	9 (27.27)	5 (4.0)	11 (3.30–36.57)	< 0.001
Injected by new syringe & needle	18 (54.55)	109 (87.2)	Reference	

^a^ Odds Ratio;

^b^ Confidence Interval

*None among male participants from cases and only two participants from controls reported ever having sex with female sex workers.

### 4.3 Sexual practices and STD symptoms

One-tenth of the cases (3/33) and controls (12/125) were unmarried. The median age at onset of sexual intercourse for males in cases was 19 year (minimum 17; maximum 45, IQR 18–33 year) and among females was 16 year (minimum 13; maximum 20, IQR 14–17 year). The median age at onset of sexual intercourse in controls among males was 19 year (minimum 12; maximum 35, IQR 17–23 year) and that in females was 18 year (minimum 14; maximum 21, IQR 16–19 year). Data on sexual practices and injection exposure during treatment seeking are presented in Table 2. No significant difference was observed between cases and controls on self-reported STD symptoms experienced over the last year except for anal ulcers (7% in cases compared to 1% in controls). Overall, very few cases and controls (< 3%) reportedly experienced genito-ulcerative or genito-secretory disease symptoms.

### 4.4 Sexually transmitted infection biomarkers and substance use

None of the participants was sero-reactive for syphilis. Table 3 presents the distribution of cases and controls across HBsAg and HSV-2 IgG without significant difference between groups. Two cases and one control could not be tested for HSV-2 IgG as serum specimens were inadequate. A striking difference in exposure to hepatitis C (indicated by the presence of HCV Ab) was identified between cases (28/33; 85%) and controls (70/125; 56%). Eighty-seven percent of people living with HIV (PLHIV cases), and 72% of controls, who were HCV sero-reactive showed the presence of HCV RNA.

**Table 3:** Unsafe injecting and sexual exposure-related biomarkers.

Characteristics	Cases	Controls	OR^a^ (95% CI^b^)	p-value
	n (%)	n (%)		
**HCV antibody**				
Sero-reactive	28 (84.8)	70 (56)	4.4 (1.5–12.1)	0.004
Sero-nonreactive	5 (15.2)	55 (44)	Reference	
**HBsAg**				
Sero-reactive	2 (6.1)	5 (4.0)	1.5 (0.28–8.36)	0.611
Sero-nonreactive	31 (93.9)	120 (96)	Reference	
**HSV-2 IgG**				
Sero-reactive	2 (6.5)	10 (8.1)	0.79 (0.16–3.78)	0.764
Sero-nonreactive	29 (93.5)	114 (91.9)	Reference	

^a^ Odds Ratio;

^b^ Confidence Interval.

About 40% of men in cases and a similar proportion in controls reported ever having a drink containing alcohol. The finding was corroborated while women participants were asked about alcohol use by their spouses. None of the participants reported ever injecting drugs for non-medicinal or recreational purposes.

### 4.5 Health seeking and HCV exposure

**Fig 2:**
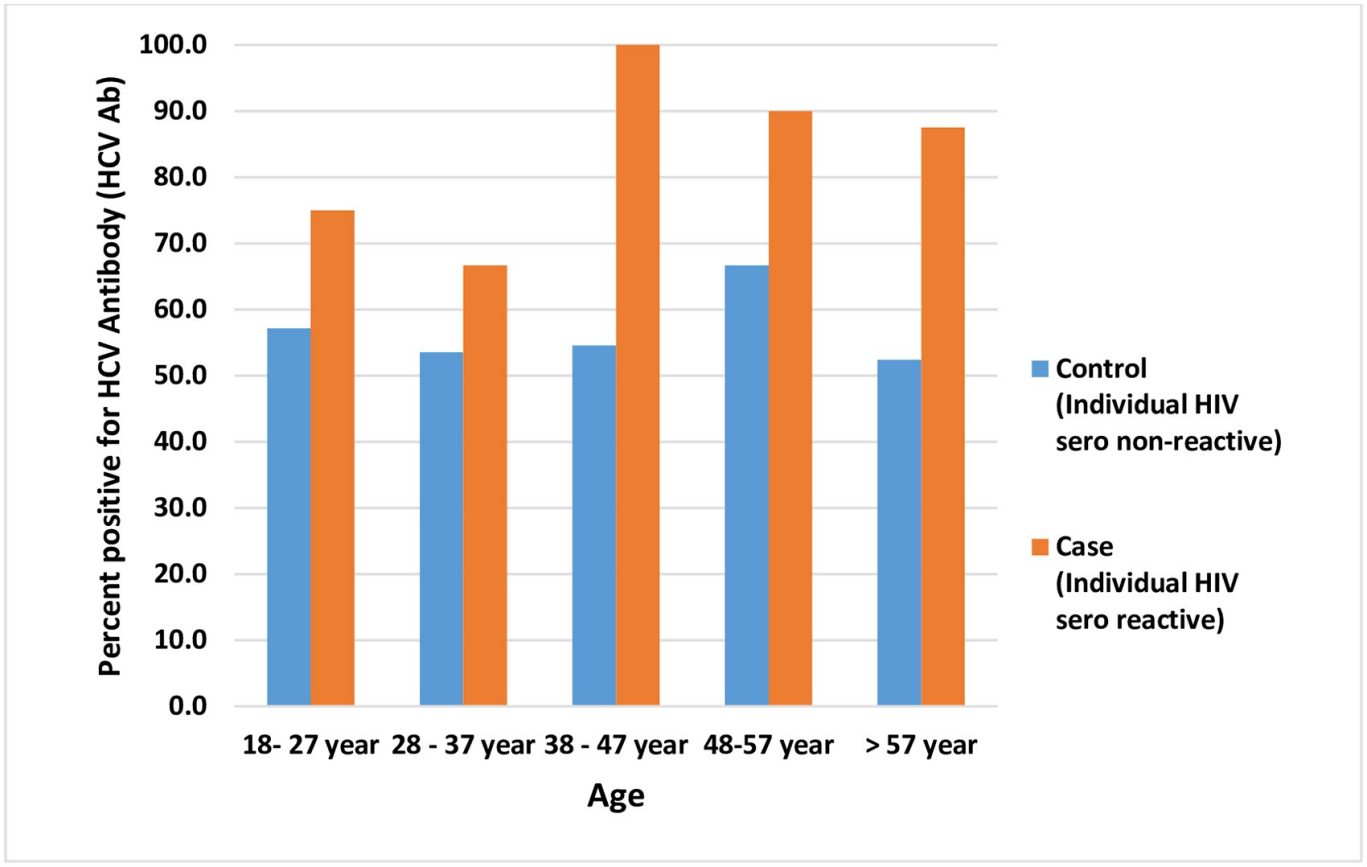
HCV sero-reactive status in HIV infected and non-infected individuals.

A significantly greater proportion of cases (32/33; 97%) sought professional help for fever, body ache, common cold and cough experienced in the last one year compared to controls; 69/125; 55% (OR 25.97; 95% CI 3.44–196; p = 0.002); plausibly indicating more illness experiences in them. We also compared cases (PLHIV) against controls regarding receipt of intravenous fluid and intramuscular injection during treatment-seeking within the last five years (Table 2), which turned out to be significantly different (97% vs 64% and 94% vs 63% respectively). This difference could be explained by the greater proneness of PLHIV to fall sick compared to controls. To investigate unsafe injection exposure, we sought treatment history over the last five years with specific attention to the multi-use of syringe and needle. A significantly higher proportion of cases reported being exposed to used syringe and needle compared to controls while seeking treatment (27% and 4% respectively; OR 11; 95% CI 3.3–36.5; p = < 0.001; Table 2). The bar diagram in Fig 2 depicts exposure to HCV infection across all age groups in cases and controls indicating a concerning level of transmission in the study community.

### 4.6 Blood transfusion and invasive procedures

Cases and controls did not differ significantly in experiencing ‘surgical procedures’, ‘blood transfusion’, ‘dental procedures’, ‘tattooing’, ‘skin-piercing’ and ‘tonsuring of the head’ (Table 4).

**Table 4:** Blood transfusion and invasive procedures.

Invasive procedure in the last 5 years	Case n (%)	Control n (%)	OR^a^ (95% CI^b^)	p-value
**Surgical procedure**				
Yes	3 (9.1)	14 (11.2)	0.79 (0.21–2.94)	0.728
No	30 (90.9)	111 (88.8)	Reference	
**Blood transfusion**				
Yes	1 (3.1)	2 (1.6)	1.98 (0.17–22.59)	0.581
No	32 (96.9)	123 (98.4)	Reference	
**Dental procedure**				
Yes	8 (24.24)	39 (31.2)	0.71 (0.29–1.70)	0.438
No	25 (75.76)	86 (68.8)	Reference	
**Tattooing**				
Yes	4 (12.1)	6 (4.8)	2.74 (0.72–10.32)	0.138
No	29 (87.8)	119 (95.2)	Reference	
**Skin piercing**				
Yes	1 (3)	4 (3.2)	0.94 (0.1–8.75)	0.961
No	32 (96.9)	121 (96.8)	Reference	
**Tonsuring**				
Yes	10 (30.3)	48 (39)	0.67 (0.29–1.55)	0.359
No	23 (69.7)	75 (60.9)	Reference	

^a^ Odds Ratio;

^b^ Confidence Interval.

### 4.7 Multivariate analysis

In the multivariate model, we included residential location as well as age as two attributes because they could reflect various confounders about which information could not be collected. Five other variables from univariate analyses, due to their statistical significance (< 0.1), and biologic plausibility qualified to be included in the multivariate logistic model. These were occupation, receipt of intramuscular injection in the last five years, exposure to used syringe and needle while seeking treatment, HCV Ab sero-reactive status and anal ulcer. We did not include HCV Ab as one of the explanatory variables in the multivariate model, because unsafe injection exposure rather than HCV would serve as a biologically plausible factor for HIV acquisition. We also did not include an anal ulcer as this was not a clinically established diagnosis. Adjusting for age, residential location and the rest of the three variables, the following two factors were found to be independently associated with HIV positive serostatus, a) ‘getting injected by a used syringe and needle’, and b) ‘receipt of intramuscular injection in last five years’ (Table 5). Unemployment, in both univariate and multivariate analyses, drew our attention towards an indicative association.

**Table 5:** Factors associated with HIV infection in multivariate analysis.

Variable	Cases	Controls	AOR^a^ (95% CI^b^)	p-value
	n (%)^a^	n (%)		
**Age**				
≤ 37 year	10 (30.3)	56 (44.8)	Reference	
> 37 year	23 (69.7)	69 (55.2)	2.07 (0.79–5.37)	0.134
**Residence**				
Premganj	18 (54.5)	62 (49.6)	0.28 (0.5–1.62)	0.157
Chakmeerapur	12 (36.4)	58 (46.4)	0.14 (0.02–0.9)	0.039
Karimuddinpur	3 (9.1)	5 (4)	Reference	
**Occupation**				
Unemployed	19 (57.58)	46 (36.80)	2.10 (0.77–5.72)	0.143
Farmer	5 (15.15)	27 (21.60)	1.06 (0.25–4.48)	0.936
Non-agricultural	9 (27.27)	52 (41.60)	Reference	
**Received intramuscular injection in last five years**				
Yes	31 (94.0)	79 (63.2)	7.20 (1.48–34.88)	0.014
No	02 (6.0)	46 (36.8)	Reference	
**Syringe & needle used while receiving intramuscular injection during the last five years**				
Didn’t notice	6 (18.18)	11 (8.8)	2.81(0.81–9.69)	0.1
Injected by used syringe & needle	9 (27.27)	5 (4.0)	6.61 (1.80–24.18)	0.004
Injected by new syringe & needle	18 (54.55)	109 (87.2)	Reference	

^a^ Adjusted Odds Ratio;

^b^ Confidence Interval.

### 4.9 Phylogenetic analysis

**Fig 3:**
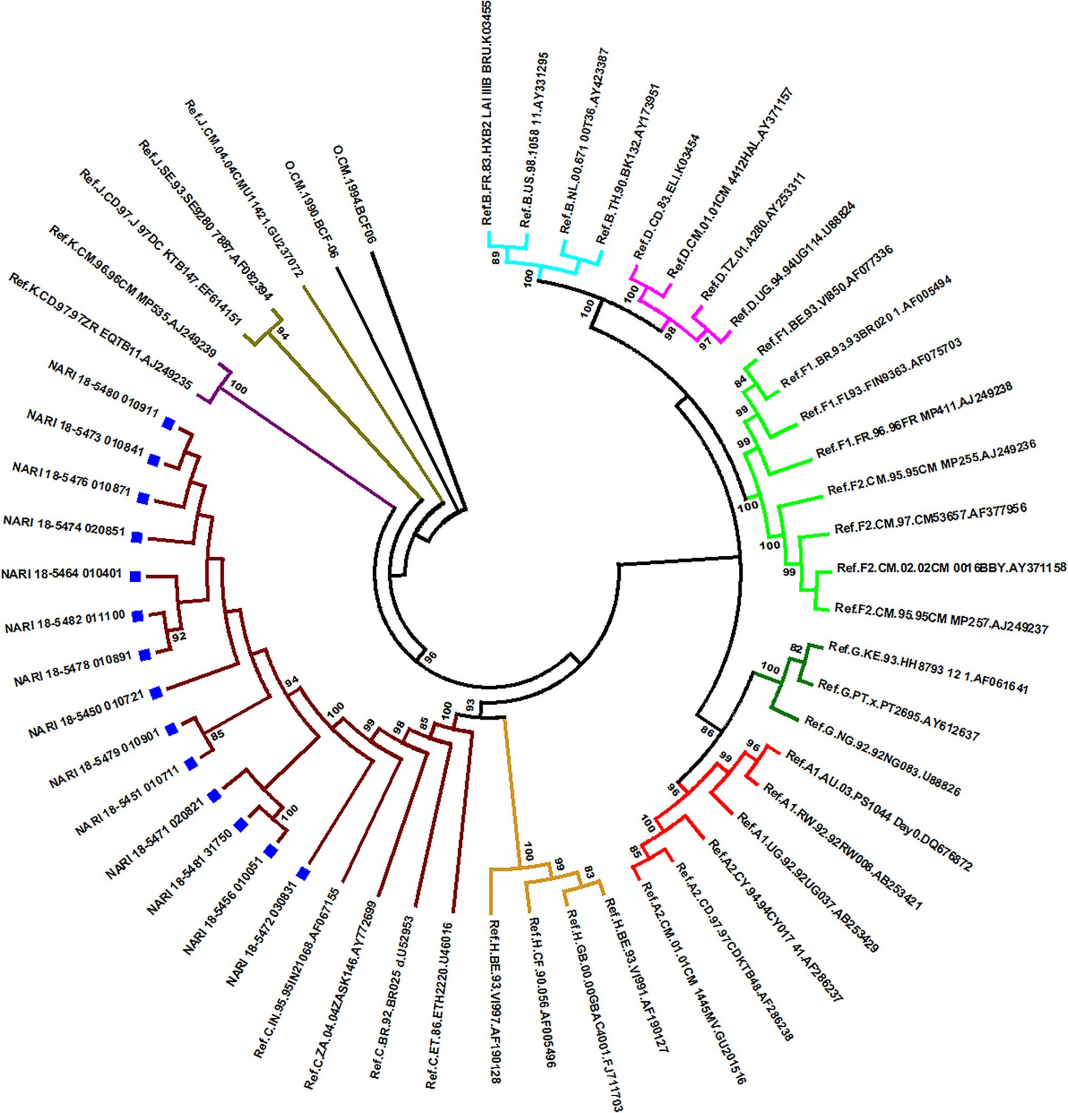
Maximum likelihood tree showing clustering of HIV-1 subtype C.

The pol gene sequences spanning HXB2 coordinates 2253–3281 from study participants (n = 14) were combined with HIV-1 subtype reference sequence alignment obtained from Los Alamos HIV sequence database (https://www.hiv.lanl.gov/content/sequence/NEWALIGN/align.html). These sequences were aligned with the help of MEGA 6 [13] and the alignment was subjected to phylogenetic analysis. Briefly, a maximum likelihood tree was constructed with the help of IQ-Tree version 1.2 [14]. The phylogenetic tree was constructed using a general time-reversible substitution model with a gamma-distributed rate of variation and a proportion of invariant sites (GTR+G+I) with 1000 bootstrap replicates. It was evident from this analysis that most of the *pol* sequences were matching best with Indian HIV-1 C subtype sequences from India. All sequences were monophyletic and had a common ancestor (Fig 3).

We identified factors associated with HIV outbreak in select locations of Bangarmau block of the study district. The way of living in these locations is mostly agrarian. Moreover, we detected a concomitant spread of HCV in the study population; 85% of the PLHIV and 56% of the controls (HIV sero non-reactive) had evidence of exposure to hepatitis C infection. In addition to these findings, secondary data analysis during the present investigation revealed a significantly rising trend of HIV among ICTC attendees in the district of Unnao during 2015–2018.

Countries in Asia have recorded HIV transmission due to sharing of injection equipments during drug use as well as faulty injection practices in therapeutic settings. For example, the Larkana district in the northwestern Province of Sindh in Pakistan witnessed the first outbreak of HIV in 2003 among PWID in whom HIV rose from 0.5% to 10% [15]. In 2016, the city of Larkana experienced spread of HIV among dialysis patients [16]. In the latter instance, information shared by a patient about his own HIV status prompted subsequent investigations.

Noticeably, between the aforementioned two events of HIV detection in Larkana, the district of Gujrat in the Punjab province of Pakistan identified spread of HIV through community based health camps [17]. Later in 2018, another HIV outbreak was reported from a village near Kot Momin, Sargodha situated in Central Punjab of Pakistan, where 1.29% of the inhabitants of the village were found to be HIV infected. Use of contaminated injection equipment by a quack doctor was implicated for this outbreak. Six months later, a survey in general population in the same village recorded 13% HIV prevalence [18]. In the recent past, a concerning level of spread of HIV was noticed during a screening campaign in Larkana. Of the 700 HIV positive cases detected among 26000 screened, 600 were under five children. The World Health Organization (WHO) declared this event as grade II emergency. Unsafe injection during treatment, contaminated blood transfusion and male circumcision with unsterile equipment were found to be the drivers of this spread [19].

Despite lack of injection drug use, presence of HCV Ab in high proportion among study participants in the current investigation was striking. Eighty Five percent of the PLHIV in this study had presence of HCV Ab, which was higher than that in controls (56%). This could be explained by greater illness experiences of PLHIV and consequent exposure to unsafe injecting during treatment seeking. Exposure to HCV among controls (56%) was also of concern and further indicated unsafe injecting in therapeutic setting (Table 3) in the local community. Worth noting here is that HCV rarely spreads through sex [20, 21] and a systematic review has documented HCV prevalence in general population in India to be ranging from 0.44% to 0.88% [22].

The finding that a significantly higher proportion of cases (27%) reported being injected by a syringe and needle already used on others compared to controls (4%), and the aforementioned evidence around HCV ruled in the possibility of unsafe injecting as a potential route of entry of HIV as well in the study community. Availability of auto-disabled syringes and needles, empowerment of the local communities and effective regulatory practices across private and public health care settings in the study area would serve as important intervention measures in such settings.

Literature search during the present investigation revealed that some of the tertiary care centers in Uttar Pradesh had recorded increasing detection of HCV among hospital attendees [23–25]. A medical institution, in the capital city of Lucknow, even highlighted that a high proportion of PLHIV (286/350; 82%) reported receiving medication through unsterile syringes and needles [26]. More than 80% of the respondents in this investigation were from rural areas.

Another probable source of HCV infection among cases and controls, all of whom were aged ≥18 year, could be contaminated blood or blood product transfusion as the units supplied from licensed blood banks in India are being screened for HCV since 2001 [27]. However, it was reassuring that no significant difference existed between cases and controls in terms of receiving blood transfusion nor with regard to invasive surgical procedures.

Our findings have close similarity to a recent iatrogenic HIV outbreak in rural Cambodia. In the Battambang province in northwest Cambodia, the family of an index HIV case, who also suffered from tuberculosis, alleged that the infection in the index case and two other family members, who became infected with HIV during the same period, were linked to medical injections received from an unlicensed health practitioner. While as high as 78% of the HIV infected individuals in this outbreak were found to be co-infected with HCV [28], in Unnao, 85% of the PLHIV had evidence of exposure to HCV infection. These two studies reveal an aspect of HCV co-infection in HIV cohorts in the Asia Pacific region, which as yet was known to range from 4% to 43% [29].

Cases ever infected with HCV, across different age groups in our study varied from 67% to 100% and in controls it ranged from 52% to 67% (Fig 2). This indicates a widespread looming risk posed to young, adults and elderly in the study community.

The arguments, presented above, raise the possibility of an iatrogenic transmission of HIV and HCV in our study setting. Discourses on the three recognized patterns of HCV epidemic [30], and evidence of fairly even presence of HCV exposure across different age groups of the study participants lend further support to this assertion. Finally, we draw attention towards the potential spread of HIV in population in other parts of Unnao as well because a rising trend of HIV was detected among ICTC attendees in the district.

We maintain that contribution of unemployment and resulting vulnerability to risk exposure during treatment seeking from settings with poor infection control could not be ruled out in our investigation. The United Nations Development Program (UNDP) ranked Unnao on 64^th^ position among erstwhile 70 districts in the state of UP on deprivation index, which is a composite indicator of income, health and education [31]. This observation has relevance to the current investigation, as HIV is known to thrive best in the settings of poverty and under development.

The current investigation was limited by being observational in nature. We interviewed the cases and controls to record exposures to potential risk factors such as unsafe sex and injection practices through recall. Socially desirable responses around these issues and recall ability were the potential sources of bias. We therefore incorporated tests for various biomarkers in our study. Such diversity in investigation approach allowed us to triangulate the findings and draw unyielding inferences. Significantly higher proportion of cases with unsafe injection exposure during treatment compared to controls over the last five years indicated that such practices might have played a role in HIV transmission in the study setting. The monophyletic clustering of HIV sequences from cases with high homology supported that they had a common ancestry. However, the current investigation was not equipped to link this ancestry with any specific source of infection.

## 6. Conclusions

We conclude that ‘experiencing unsafe injecting’ in care settings was strikingly high among PLHIV compared to those who did not contract HIV. The role of such unsafe injecting cannot therefore be ruled out as a factor associated with HIV transmission in the study area. Disparately high presence of HCV Ab among HIV sero-reactive individuals compared to controls and current knowledge about HCV transmission also lend support to our assertion. We underline the need for ensuring safe injection practices in therapeutic settings in the study area at the earliest. Making auto-disabled syringes and needles available in all care settings, empowerment of the local community and effective regulatory practices will play pivotal roles in this regard.

## Supporting information

S1 File (PDF)

## References

[1] Simoes EA, Babu PG, John TJ, Nirmala S, Solomon S, Laxminarayana CS, et al Evidence for HTLV-III infection in prostitutes in Tamil Nadu (India). Indian J Med Res 1987; 85: 335–338. 3623641

[2] AIDS Strike Calcutta. The Telegraph. 19th August 1990. (accessed through dspace.wbpublibnet.gov.in › xmlui › bitstream › handle).

[3] Fox R. Red light on Calcutta, health via school in Andhra Pradesh. Lancet 1994; 344:1038 10.1016/s0140-6736(94)91705-1 7934442

[4] World Health Organization. The HIV/AIDS Pandemic. 1993 Overview. Geneva: World Health Organization, 1993.

[5] Naik T.N., Sarkar S., Singh H.L., Bhunia S.C., Singh Y.I., Singh P.K., et al (1991). Intravenous drug users-a new high-risk group for HIV infection in India. *AIDS* 5 (1): 117–118. 10.1097/00002030-199101000-00026 2059356

[6] Sarkar S, Das N, Panda S, Naik TN, Sarkar K, Singh BC, et al Rapid spread of HIV among injecting drug users in northeastern states of India. Bull Narc 1993; 45 (1): 91–105. 8305909

[7] Sgaier SK, Claeson M, Gilks C, Ramesh BM, Ghys PD, Wadhwani A, et al Knowing your HIV/AIDS epidemic and tailoring an effective response: how did India do it? Sex Transm Infect. 2012; 88(4): 240–249. 10.1136/sextrans-2011-050382 22510332 PMC3351854

[8] Panda S, Roy T, Pahari S, Mehraa J, Sharma N, Singh G, et al Alarming epidemics of human immunodeficiency virus and hepatitis C virus among injection drug users in the northwestern bordering state of Punjab, India: prevalence and correlates. International Journal of STD &AIDS 2014; 25(8):596–606. 10.1177/0956462413515659 24352120

[9] National AIDS Control Organization (NACO 2015), Government of India. National Integrated Biological and Behavioral Surveillance (IBBS), High-Risk Groups 2014–2015, New Delhi.

[10] Census 2011. "Census of India Website: Office of the Registrar General & Census Commissioner, India". www.censusindia.gov.in. accessed on 2019-01-15.

[11] Census 2011. "Census of India Website: Office of the Registrar General & Census Commissioner, India"http://censusindia.gov.in/2011census/dchb/0925_PART_B_DCHB_UNNAO.pdf. accessed on 2019-01-15

[12] Chaturbhuj DN, Nirmalkar AP, Paranjape RS, Tripathy SP (2014). Evaluation of a Cost-Effective In-House Method for HIV-1 Drug Resistance Genotyping Using Plasma Samples. PLOS ONE 9(2): e87441 10.1371/journal.pone.0087441 24533056 PMC3922725

[13] Tamura K, Stecher G, Peterson D, Filipski A, Kumar S. MEGA6: Molecular Evolutionary Genetics Analysis version 6.0. Molecular biology and evolution. 2013; 30(12):2725–9. 10.1093/molbev/mst197 24132122 PMC3840312

[14] Trifinopoulos J, Nguyen LT, von Haeseler A, Minh BQ. W-IQ-TREE: a fast online phylogenetic tool for maximum likelihood analysis. Nucleic acids research. 2016;44(W1): W232–5. 10.1093/nar/gkw256 27084950 PMC4987875

[15] Shah SA, Altaf A, Mujeeb SA, Memon A. An outbreak of HIV investigation among injection drug users in a small town in Pakistan: potential for national implication. Int J STD AIDS 2004;15:209. 10.1258/095646204322916713 15038874

[16] Altaf A, Pasha S, Vermund S, Shah S. A second major HIV outbreak in Larkana, Pakistan. Journal of Pakistan Medical Association 2016; 66(12): 1510–11. 27924955 PMC10767707

[17] Ansari J, Salman M, Safdar RM, Ikram N, Mahmood T, Zaheer H. HIV/AIDS outbreak investigation in Jalalpur Jattan (JPJ), Gujrat, Pakistan. Journal of Epidemiology and Global Health 2013; 3: 261–268. 10.1016/j.jegh.2013.06.001 24206797 PMC7320408

[18] Zaid M, Afzal SM. HIV outbreak in Pakistan. The Lancet Infectious Diseases 2018; 18(6): 601 10.1016/S1473-3099(18)30281-0 29856353

[19] Ahmed A, Hashmi FK, Khan GM. HIV outbreaks in Pakistan. The Lancet Infectious Diseases 2019; 6(7): 418 10.1016/S2352-3018(19)30179-1 31204244

[20] Terrault NA, Dodge JL, Murphy EL, Tavis JE, Kiss A, Levin TR, et al Sexual transmission of hepatitis C virus among monogamous heterosexual couples: the HCV partners study. Hepatology. 2013; 57(3):881–889. [10.1002/hep.26164 ] 23175457 PMC4384338

[21] Dodge JL, Terrault NA. Sexual transmission of hepatitis C: A rare event among heterosexual couples. J Coagul Disord. 2014; 4(1):38–39. 26617979 PMC4659344

[22] Goel A, Seguy N, Aggarwal R. Burden of hepatitis C virus infection in India: A systematic review and meta-analysis. J Gastroenterol Hepatol. 2019; 34(2):321–329. 10.1111/jgh.14466 30176181

[23] Shantanu P, Amita J Sankhwar S, Usman K, Narayan P, Saha D, et al Prevalence of Hepatitis B and C virus among patients on hemodialysis in Lucknow, Uttar Pradesh. Clinical Epidemiology & Global Health 2 2014: 19–23. 10.1016/j.cegh.2013.03.001

[24] Khatoon R, Jahan N. Assessment of seroprevalence of hepatitis C virus-specific antibodies among patients attending hospital of semi-urban North India using a rapid qualitative in-vitro diagnostic test. Ann Trop Med Public Health 2017; 10:199–204.

[25] Agarwal L, Singh AK, Agarwal A, Singh RP. Incidental detection of hepatitis B and C viruses and their coinfection in a hospital-based general population in tertiary care hospital of Uttar Pradesh. J Family Med Prim Care 2018; 7:157–61. 10.4103/jfmpc.jfmpc_196_16 29915751 PMC5958559

[26] Wal N, Venkatesh V, Agarwal GG, Kumar A, Tripathi AK, Singh M et al Unsafe injections: a potential risk for HIV transmission in India. *Biomedical Research*; 23 (3): 390–394.

[27] Ministry of Health & Family Welfare, Government of India; National Health Mission. National Action Plan Combating Viral Hepatitis in India 2019.

[28] Rouet F, Nouhin J, Zheng D, Roche B, Black A, Prak S, et al Massive Iatrogenic Outbreak of Human Immunodeficiency Virus Type 1 in Rural Cambodia, 2014–2015 Clinical Infectious Diseases 2018;66(11): 1733–1741, 10.1093/cid/cix1071 29211835 PMC5963970

[29] Martinello M, Amin J, Matthews GV and Dore GJ. Prevalence and disease burden of HCV coinfection in HIV cohorts in the Asia Pacific Region: a systematic review and meta-analysis. AIDS Rev. 2016 Apr-Jun;18(2):68–80. 27196354

[30] World Health Organization. Guidelines on hepatitis B and C testing. Geneva; February 2017.

[31] UNDP. Human Development Reports– 2007. United Nations Development Programme. http://hdr.undp.org/sites/default/files/india_uttar_pradesh_2007.pdf [accessed last on 25th October 2019].

