# Supplementary material for: Unsterile injection equipment associated with HIV outbreak and an extremely high prevalence of HCV—A case-control investigation from Unnao, India
Source: PLoS One. 2020 Dec 4;15(12):e0243534. doi: 10.1371/journal.pone.0243534 (PMC7717531; doi:10.1371/journal.pone.0243534)
Supplement: S1 File — (PDF) [file pone.0243534.s001.pdf]

पहचान क्रमांक						

गाँव व्यक्ति

Epidemiological and Laboratory Investigation in rural Uttar Pradesh, India: a case–control study

एक साक्षात्कार अनुसूची
One on one interview schedule

जरूरी अनुस्मारक
IMPORTANT REMINDERS

- एक साक्षात्कार की अनुसूचि का केवल एक ही व्यक्ति के लिए उपयोग किया जा सकता है|
  One interview schedule is to be used for one individual only
- अगर किसी ने जवाब नहीं दिया(code 99) तो दुसरे प्रश्न की ओर जाएँगे|
  In case of 'no response' (code 99) to any question, the interviewer should proceed to the next question
- सही उत्तर पर गोला लगाएँ (√मत कीजिये) |
  Circle the codes (not tick) against each question as appropriate
- प्रश्न छोड़ने की सूचना ध्यान से पढ़िए क्योकि कुछ प्रश्न पूछने की जरुरत नहीं होगी।यह पिछले प्रश्न की जवाब पर निर्भर होगा।
  Some questions need not to be asked based on the preceding response-pay attention to skip questions
- कृपया ध्यान दें कि कुछ प्रश्नों के विकल्पों को पढ़ने की आवश्यकता है।
  Please note that for some questions options need to be read out.

परिचय और पात्रता मापदंड
Introduction and Section on checking eligibility criteria

मेरा नाम ______________________________ है | मैं_______________________(विभाग) के लिए काम करता / करती हूँ |हम स्वास्थ्य सम्बंधित सम्स्याओ के बारे में मालूम करने के लिए ______________________ यहाँ पर लोगों का साक्षात्कार कर रहे हैं | आपके जवाब से हमें स्वास्थ्य सम्बंधित सम्स्याओ के बारे में समझने में आसानी होगी, और हम उस पर उपयुक्त कदम उठा सकते है |

"My name is... I'm working for ……….. (Department). We're interviewing people here in …………….............. (Name of the locality) in order to find out various health related risks. Your response will help me to understand health issues better and develop appropriate interventions.

1. आपकी उम्र क्या है?<br>How old are you (in completed year)? [__|__]<br>2. जन्म तारीख<br>Date of birth: ------------------------------------<br>3. जन्म तारीख का दाखला<br>Source of Evidence: --------------------------------<br>(for date of birth)	**अगर उम्र १८ साल से कम है तो प्रतिभागी का धन्यवाद् करके साक्षात्कार वही समाप्त कीजिये |**<br>If Age < 18 (minor) thank the respondent and terminate the interview
4. उत्तरदाता का लिंग<br>Sex of the respondent	**पुरुष** MALE 1<br>**स्त्री** FEMALE **2**<br>**हिजड़ा** TRANSGENDER **3**

पहचान क्रमांक						

गाँव व्यक्ति

गोपनीयता और सहमती

Confidentiality and consent

मैं आपको कुछ सवाल पूछ ने जा रहा / रही हूँ | आपका जवाब पूरी तरह गोपनीय है | आपका नाम इस फ़ॉर्म पर कहीं भी नहीं लिखा जाएगा और कभी भी आप के द्वारा बताई गई किसी भी जानकारी से नहीं जोडा जाएगा। आपको किसी भी सवाल का जवाब देना नहीं है जिसका जवाब आप नहीं देना चाहते।आप किसी भी समय इस साक्षात्कार को छोड़ सकते हैं I हालांकि, इन सवालों के आपके ईमानदार उत्तर हमें समझने में मदद करेंगे कि लोग क्या सोचते हैं और अपने स्वास्थ्य के बारे में क्या कहते हैं I हम इस मूल्यांकन प्रश्नावली का जवाब देने में आपकी सहायता मांग रहे है। साक्षात्कार में लगभग बीस मिनट लगेंगे। क्या आप इस अध्ययन में भाग लेने के लिए तैयार होंगे?

सहमति निर्धारित करने के लिए सहमति पत्र (आईसीएफ) का उपयोग करें और आईसीएफ पर उपरोक्त पहचान कोड भी लिखें।

"I'm going to ask you some questions. Your answers are completely confidential. Your name will not be written anywhere on this form and will never be used in connection with any of the information you tell me. You do not have to answer any question that you do not want to answer, and you may end this interview at any time you want to. However, your honest answers to these questions will help us understand what people think, and say about their health. We would greatly appreciate your help in responding to this assessment questionnaire. The interview will take approximately twenty minutes.

Would you be willing to participate? Use INFORMED CONSENT FORM (ICF) to obtain consent and write the identification code as above on the ICF as well.

सहमती पत्र का प्रकार Type of consent obtained (Mark √ as appropriate):

1. Oral ☐ **2. Signature** ☐ **3. Left thumb impression** ☐

1. तारीख (Date)		
2. स्थान (नाम) Locality (interviewing place) (name)		
3. साक्षात्कार कर्ता Interviewer	नाम: ------------------------------ Name: ------------------------------------	हस्ताक्षर: ---------------- Signature: ---------------
4. पर्यवेक्षक/ डाटा एंट्री ऑपरेटर (जिन्होंने भरी हुई प्रश्नावली की गुणवत्ता की जांच की और उस में आवश्यक परिवर्तन किया) Supervisor/Data entry operator (who checked the quality of filled in questionnaire)	नाम: ------------------------------ Name: ------------------------------------	हस्ताक्षर: ---------------- Signature: ---------------
5. परिणाम क्रमांक (पर्यवेक्षक के द्वारा लिखा गया) **गोला लगाइये।** Result code for the interview (encircle)	**Result codes:** Completed – 1 Partially completed – 2 Refused – 3	

पहचान क्रमांक						

गाँव व्यक्ति

भाग 0: सामान्य सवाल

Section 0: General questions

**अनुक्रमांक No.**	**प्रश्न Questions and filters**	**कोडिंग के प्रकार Coding categories**	**प्रश्न क्रमांक पर जाइये Skip to**
**Q 001**	**इस जगह पर कितने समय से आप रह रहे हैं?**<br>**(महीनो या साल में दर्ज कीजिये)**<br>How long have you been living at this place?	--<br>--	
Q 002	**इस जगह पर बच्चों की आम स्वास्थ्य समस्या क्या है?**<br>**(जैसा कि कहा गया वैसे लिखे)**<br>What is the commonest health problem of children here at this place?	--<br>--	
Q 003	**इस जगह पर काम कर रहे प्रौढ़ पुरुषों की आम स्वास्थ्य समस्या क्या है?**<br>**(जैसा कि कहा गया वैसे लिखे)**<br>What is the commonest health problem of working adult men here at this place?	--<br>--	
Q 004	**इस जगह पर काम कर रहे प्रौढ़ महिलाओं की आम स्वास्थ्य समस्या क्या है?**<br>**(जैसा कि कहा गया वैसे लिखे)**<br>What is the commonest health problem of adult female here at this place?	--<br>--	
Q 005	**इस जगह पर 60 साल या उससे अधिक उम्र के लोगों की आम स्वास्थ्य समस्या क्या है?**<br>**(जैसा कि कहा गया वैसे लिखे)**<br>What is the commonest health problem of people above 60 years or older here at this place?	--<br>--	

पहचान क्रमांक						

गाँव व्यक्ति

भाग १:सामाजिक जनसांख्यिकीय विशेषतायें

Section 1: Socio-demographic characteristics

अनुक्रमांक<br>No.	प्रश्न<br>Questions and filters	कोडिंग के प्रकार<br>Coding categories	प्रश्न क्रमांक पर जाइये Skip to
Q 101	उत्तरदाता का लिंग<br>Record sex of the respondent<br>(एक ही जवाब को गोला लगायें)	पुरुष MALE 1<br>स्त्री FEMALE 2<br>किन्नर/हिजड़ा TRANSGENDER 3	
Q 102	क्या आप कभी भी स्कूल गए हो?<br>Have you ever attended school?<br>(जैसे बताया गया वही लिखो)	हाँ YES 1<br>नहीं NO 2<br>जवाब नहीं दिया NO RESPONSE 3	यदि 'नहीं' तो अनुक्रमांक 104 पर जाए<br>If 'no' go to Q 104
Q 103	कोन सी कक्षा पास की है?<br>What level of schooling did you complete? (Examination passed)<br>(पढ़कर सुनाये |एक ही जवाब को गोला करे)	प्राथमिक स्कूल से पहले BEFORE PRIMARY 1<br>६ वी कक्षा तक PRIMARY SCHOOL (6th passed) 2<br>८ वी कक्षा तक LOWER SECONDARY (8th) 3<br>१० वी कक्षा तक MIDDLE SECONDARY (10th) 4<br>१२वी कक्षा तक HIGHER SECONDARY (12th) 5<br>बैचलर डिग्री या उच्च BACHELOR DEGREE OR HIGHER 6<br>अनौपचारिक शिक्षा NON FORMAL EDUCATION 7<br>कोई जवाब नहीं दिया NO RESPONSE 99	
Q 104	क्या आप अपने घर से काम के लिए ३ महीने से अधिक समय के लिए दूर रहे हो?<br>Did you ever stay away from your home for work at a stretch for a period of 3 months or more?<br>(जैसे बताया गया वही लिखो)	हाँ YES 1<br>नहीं NO 2<br>जवाब नहीं दिया NO RESPONSE 99	यदि 'नहीं' तो अनुक्रमांक 106 पर जाए<br>If 'no' go to Q106
Q105(A)<br><br>Q105(B)	यदि हाँ, तो कोन सी जगह थी ? What was the place?<br>A] शहर City<br>B] गाँव Village<br>C] नगर Town<br>(जैसे बताया गया वही लिखो)<br>आप वहाँ थे, क्या आपके पति/ पत्नी आपके साथ थे?<br>Did you have your husband/ wife then along with you?<br>(जैसे बताया गया वही लिखो)	A]---<br>B] ---<br>C] ---<br><br>हाँ YES 1<br>नहीं NO 2<br>तब में शादीशुदा नहीं था I WAS NOT MARRIED THEN 3<br>जवाब नहीं दिया NO RESPONSE 99	

Q 106	क्या आप यहाँ पर काम के वजह से घर से दूर रुके है ?<br>Are you now living here in this place/locality away from your home for work?<br>**(पढ़कर सुनाये |एक ही जवाब को गोला करे)**	हाँ YES 1<br>नहीं मेरा घर यहाँ है | NO, MY HOME IS HERE 2<br>नहीं में यहाँ रिश्तेदार के साथ रहता हूँ| NO, I AM STAYING AT MY RELATIVE'S PLACE 3<br>नहीं में यहाँ पढाई करने के लिए ठहरा हूँ| NO, I AM HERE FOR MY STUDIES 4<br>जवाब नहीं दिया NO RESPONSE 99	**यदि 'नहीं' तो अनुक्रमांक 108(A)पर जाए**<br>**If 'no' go to Q108 (A)**
Q 107	यदि आप यहाँ पर काम की वजह से घर से दूर रुके हो, तो आप इस जगह पे कब से हो?<br>How long have you lived in here?<br>**(जैसे बताया गया वही लिखो)**	________ माह से NUMBER OF MONTHS<br>________ वर्षों से NUMBER OF YEARS<br>पता नहीं DON'T KNOW 88<br>जवाब नहीं दिया NO RESPONSE 99	
Q108(A)	क्या अपने कभी शादी की है?<br>Did you ever get married?<br>**(जैसे बताया गया वही लिखो)**<br>I am going to ask you in details about your partner.<br>**मैं आपको आपके साथी के बारे पूछने जा रहा हु।**	हाँ YES 1<br>नहीं NO 2<br>जवाब नहीं दिया NO RESPONSE 99	**यदि 'हाँ' तो अनुक्रमांक 108(B) और 108 (C) पर जाए**<br>**यदि 'नहीं' तो अनुक्रमांक 108(D) और 108 (E)** If no' go to Q 108 (D) &108 (E)<br>If 'yes' go to Q 108 (B) &
Q108(B)	If you ever got married<br>अपने कभी शादी की है तो<br>Read out and explore<br>**(पढ़कर सुनाये |एक ही जवाब को गोला करे)**	शादीशुदा और पति/ पत्नी के साथ रह रहे है| MARRIED & LIVES WITH SPOUSE 1<br>शादीशुदा है परन्तु पति/ पत्नी काम के वजह से दूर रह रहा है MARRIED & SPOUSE AWAY FOR JOB 2<br>शादी के बाद अलग रह रहे है MARRIED BUT If SEPARATED 3<br>शादी के बाद अलग हो चुके है | MARRIED AND DIVORCED 4<br>जोड़ीदार का मृत्यु हो चूका है| WIDOWER 5<br>कोई जवाब नहीं दिया NO RESPONSE 99	
Q108(C)	If you ever got married and stay with spouse, what does/did he/she do to earn money?<br>अपने कभी शादी की है तो, आपका पति /पत्नी कमाने के लिए क्या काम करता/ करती हैं/थे?<br>**(जैसे बताया गया वही लिखो)**	-----------------------<br>कोई जवाब नहीं दिया| NO RESPONSE 99	

पहचान क्रमांक						

गाँव व्यक्ति

Q108(D)<br><br><br><br>Q108(E)	If you never got married<br>अब तक शादी नहीं हुई है तो<br>(जैसे बताया गया वही लिखो)<br><br>If you never got married but stay with partner, what does/did he/she do to earn money?<br>अब तक शादी नहीं हुई है मगर जोड़ीदार साथी के साथ रह रहे है, तो आपका साथी कमाने के लिए क्या काम करता हैं/था?<br>(जैसे बताया गया वही लिखो)	अब तक शादी नहीं हुई मगर जोड़ीदार साथी के साथ रह रहे है। UNMARRIED BUT STAYING WITH PARTNER 1<br>कोई जवाब नहीं दिया NO RESPONSE 99<br><br>------------------------<br>कोई जवाब नहीं दिया। NO RESPONSE 99	
Q 109	आप कमाने के लिए क्या काम करते हो?<br>What do you do to earn money?<br>(जैसे बताया गया वही लिखो)	------------------------<br>कोई जवाब नहीं दिया। NO RESPONSE 99	अगर पैसा कमाने के लिए काम नहीं करते है तो अनुक्रमांक 201 पर जाइये ।<br>If does not work to earn money go to Q 201
Q 110	पिछले महीने मे आपने कितने दिनो तक काम किया?<br>In the past one month, how many days did you work?<br><br>(पढ़कर सुनाये ।एक ही जवाब को गोला करे)	महीने मे पूरे दिन काम किया WORKED ALMOST ALL DAYS 1<br>ज्यादा से ज्यादा दिन काम किया WORKED FOR MOST DAYS 2<br>आधे महीने तक काम किया WORKED ABOUT HALF THE MONTH 3<br>लगभग काम नहीं मिला WAS MOSTLY UNEMPLOYED 4<br>बेरोजगार UNEMPLOYED 5<br>मालूम नहीं DON'T KNOW 88<br>कोई जवाब नहीं दिया NO RESPONSE 99	
Q 111	आप को काम के लिए अपने घर से हप्ते भर के लिए या उससे ज्यादा दिनोंके लिए घर से दूर रहना पड़ता है क्या ?<br>(जैसे बताया गया वही लिखो)<br>In your present work do you at times need to stay away from your residence for a week or more?	हाँ YES 1<br>नहीं NO 2<br>जवाब नहीं दिया NO RESPONSE 99	
Q 112	आप महीने में लगभग कितने रुपये कमाते हो? (जैसे बताया गया वही लिखो)<br>On an average how much do you earn (in Rupees) in a month?	---------------- रू.<br>कोई जवाब नहीं दिया NO RESPONSE 99	

पहचान क्रमांक						

गाँव व्यक्ति

भाग २. स्वास्थ्य सम्बंधित
Section 2: Health issues

**अनुक्रमांक No.**	**प्रश्न Questions and filters**	**कोडिंग के प्रकार Coding categories**	**प्रश्न क्रमांक पर जाइये Skip to**
Q 201	क्या आप को **पिछले एक साल** में कोई बीमारी हुई थी जिस के लिए आप किसी डॉक्टर के पास गए थे?<br>Did you experience any kind of illness / sickness in the LAST ONE YEAR for which you had to take professional help?	हाँ YES 1<br>नहीं NO 2<br>जवाब नहीं दिया NO RESPONSE 99	**यदि 'नहीं' तो अनुक्रमांक 203 पर जाए**<br>**If 'no' go to Q. 203**
Q 202	A] **पिछले एक साल** में आपको कौनसी बीमारी हुई थी?<br>What was the illness/ sickness?<br>**(जैसे बताया गया वही लिखो)**<br>**B] पिछले एक साल** में आप बीमारी के लिए किस के पास गए थे ? From whom did you seek medical advice for the illness you had in the LAST ONE YEAR?<br>डॉक्टर कौन से चिकित्सा प्रणाली के थे?<br>System practiced by the doctor?<br>**(पढ़कर सुनाये |एक ही जवाब को गोला करे)**<br><br>C] इलाज के लिए सुई लगाई थी ?<br>Were injections given?	A] ____________________<br><br>B] अंग्रेजी दवा देने वाले / अलोपथी ALLOPATHY 1<br>मीठी दवा देने वाले / होम्योपैथी HOMEOPATHY 2<br>आयुर्वेदा AYURVEDA 3<br>यूनानी UNANI 4<br>झोला छाप डाक्टर QUACK 5<br>पता नहीं DON'T KNOW 88<br><br>C] हाँ YES 1<br>नहीं NO 2<br>जवाब नहीं दिया NO RESPONSE 99	
Q 203	**पिछले पांच सालों** में क्या आप को नसों में सुई से कोई दवाई दी गयी है?<br>Did you ever receive fluid in your vein as treatment of any illness you had within the LAST 5 YEARS?<br>**(जैसे बताया गया वही लिखो)**	हाँ YES 1<br>नहीं NO 2<br>जवाब नहीं दिया NO RESPONSE 99	
Q 204	**पिछले पांच सालों** में क्या आप को मांस पेशी में इलाज के लिए सुई दी गयी है?<br>Did you receive injection in your muscle as treatment of any illness you had within the LAST 5 YEARS?<br>**(जैसे बताया गया वही लिखो)**	हाँ YES 1<br>नहीं NO 2<br>जवाब नहीं दिया NO RESPONSE 99	**यदि 'नहीं' तो अनुक्रमांक 206 पर जाए**<br>**If 'no' go to Q206**

पहचान क्रमांक						

गाँव व्यक्ति

Q 205	क्या आप को नई सुई और इंजेक्शन (**बंद पैक से खोली गयी**) दी गयी है ?<br>Was brand new syringe and needle (opened from a sealed pack) used to administer medicine to you?<br>**(जैसे बताया गया वही लिखो)**	हाँ YES 1<br>नहीं NO 2<br>देखा नहीं DID NOT NOTICE 3<br>जवाब नहीं दिया NO RESPONSE 99	
Q 206	**पिछले पांच सालों** में आप का कोई ऑपरेशन हुआ था ?<br>Have you undergone any surgical procedure within the LAST 5 YEARS?<br>**(जैसे बताया गया वही लिखो)**	हाँ YES 1<br>नहीं NO 2<br>जवाब नहीं दिया NO RESPONSE 99	
Q 207	A] **क्या पिछले पांच सालों** में आप को खून चढ़ाया गया था? Did you receive blood transfusion in LAST 5 YEARS for any reason<br>**(जैसे बताया गया वही लिखो)**<br><br>B] कारन लिखो की आपको खून क्यों चढ़ाया था।<br>**RECORD AS STATED (ask why blood transfusion was necessary?)**<br>**(जैसे बताया गया वही लिखो)**	A] हाँ YES 1<br>नहीं NO 2<br>जवाब नहीं दिया NO RESPONSE 99<br><br>B] --------------------------------------	
Q 208	**पिछले पांच सालों** में क्या आप ने अपने दातों का इलाज करवाया?<br>Did you undergo any dental procedure within LAST 5 YEARS?<br>**(जैसे बताया गया वही लिखो)**	हाँ YES 1<br>नहीं NO 2<br>जवाब नहीं दिया NO RESPONSE 99	
Q 209	A] क्या **पिछले पांच सालों** में आपने कभी भी अपने शरीर पर गोदना गुदवाया?<br>Did you ever do tattooing on your body within the LAST 5 YEARS?<br><br>B] आपने कभी भी अपने शरीर पर गोदना गुदवाया है तो, गोदना का प्रकार क्या था?<br>If you ever did tattooing on your body, what type of tattoo making did you go through?<br><br>**(जैसे बताया गया वही लिखो)**	A] हाँ YES 1<br>नहीं NO 2<br>जवाब नहीं दिया NO RESPONSE 99<br><br>B] हाँथ से पारंपरिक पद्धति अनुसार जिसमे खून कपडे से पोंछा जाता है TRADITIONALLY BY HAND AND WIPINGOFF BLOOD WITH CLOTH 1<br>बैटरी संचलित सुई का इस्तेमाल करके BATTERY OPERATED NEEDLES 2	
Q 210	A] **पिछले पांच सालों** में क्या आपने कभी अपने शरीर में किसी भी कारण छेद करवाया है?<br>Did you ever undergo skin piercing for any reason within the LAST 5 YEARS?	अ] हाँ YES 1<br>नहीं NO 2<br>जवाब नहीं दिया NO RESPONSE 99	

पहचान क्रमांक						

गाँव व्यक्ति

	B] **<u>पिछले पांच सालों</u>** में क्या आपने कभी किसी भी कारण मुंडन करवाया है?<br>Did you ever undergo tonsuring of head for any reason within the LAST 5 YEARS?	B] हाँ YES 1<br>नहीं NO 2<br>जवाब नहीं दिया NO RESPONSE 99	
Q.211(A)<br><br><br><br><br>Q.211(B)	आप बाल कटवाने के लिए क्या करते हो?<br>What do you usually do for getting your haircut?<br>**(केवल पुरुषों पढ़कर सुनाये |एक ही जवाब को गोला करे)**<br><br>आप दाढ़ी बनाने के लिए क्या करते हो?<br>What do you usually do for shaving (beard/moustache)?<br>**(केवल पुरुषों पढ़कर सुनाये |एक ही जवाब को गोला करे)**	हाँ नाई के दूकान के पास जाते है GO TO A BARBER SHOP 1<br>रास्ते पर बैठे हुए नाई के पास जाते है AVAIL ROADSIDE BARBER SERVICE 2<br>नाई घर पे आता है BARBER COMES HOME 3<br>जवाब नहीं दिया NO RESPONSE 99<br><br>हाँ नाई के दूकान के पास जाते है GO TO A BARBER SHOP 1<br>रास्ते पर बैठे हुए नाई के पास जाते है AVAIL ROADSIDE BARBER SERVICE 2<br>नाई घर पे आता है BARBER COMES HOME 3<br>जवाब नहीं दिया NO RESPONSE 99	
Q 212	क्या नाई के दूकान में हर बार नई ब्लेड का उपयोग किया जाता है?<br>Does your barber usually get a new blade every time for you every time?<br>**(केवल पुरुष एक ही जवाब को गोला करे)**	हाँ YES 1<br>नहीं NO 2<br>पता नहीं DON'T KNOW 88<br>जवाब नहीं दिया NO RESPONSE 99	
Q 213	**<u>पिछले पांच सालों</u>** में क्या आप ने किसी भी कारण दूसरे व्यक्ति के द्वारा इस्तेमाल की हुई सुई का उपयोग किया ?<br>Did you receive injection for any purpose within the LAST 5 YEARS while same injection equipment was used on different individuals?<br>**(एक ही जवाब को गोला करे)**	हाँ YES 1<br>नहीं NO 2<br>जवाब नहीं दिया NO RESPONSE 99	

पहचान क्रमांक						

गाँव व्यक्ति

भाग 3. मद्यपान और मादक द्रव्यों का सेवन से सम्बंधित प्रश्न

Section 3: About alcohol or substance use practices

मैं आपके मद्यपान /शराब और मादक द्रव्यों का सेवन से सम्बंधित कुछ सवाल पूछने जा रहा/रही हूं।कृपया शर्मिंदगी महसूस न करें| मैं आपको फिर से आश्वासन देता/देती हूं कि जो भी आप कहते हैं वह गोपनीय रहेगा|

अनुक्रमांक No.	प्रश्न Questions and filters	कोडिंग के प्रकार Coding categories	प्रश्न क्रमांक पर जाइये Skip to
Q 301	क्या आपने कभी भी मद्यपान/ शराब का सेवन किया है ?<br>Have you ever had a drink containing alcohol? **(जैसे बताया गया वही लिखो)**	हाँ YES 1<br>नहीं NO 2<br>जवाब नहीं दिया NO RESPONSE 99	**यदि 'नहीं' तो अनुक्रमांक 304 पर जाए If 'no' go to Q 304**
Q 302	जब आपने पहली बार मद्यपान / शराब का सेवन किया था तब आप की उम्र क्या थी?<br>How old were you when you first had alcohol?<br>**(जैसे बताया गया वही लिखो)**	---------- उम्र AGE IN COMPLETED YEARS 1<br>पता नहीं DON'T KNOW 88<br>कोई जवाब नहीं दिया NO RESPONSE 99<br>**(सही उत्तर का अनुमान लगाए)**	
Q 303	क्या आपने **<u>पिछले पांच सालों</u>** में मद्यपान /शराब के कारण हुए तकलीफ़ के लिए इलाज करवाया?<br>Have you received treatment for problem alcohol use within the LAST FIVE YEAR?<br>**(जैसे बताया गया वही लिखो)**	हाँ YES 1<br>नहीं NO 2<br>जवाब नहीं दिया NO RESPONSE 99	
Q 304	क्या आपने कभी भी सिर्फ मनोरंजन के लिए नशीली पदार्थो का इस्तेमाल किया था ?<br>Did you ever take drugs for recreation?<br>**(जैसे बताया गया वही लिखो)**	हाँ YES 1<br>नहीं NO 2<br>जवाब नहीं दिया NO RESPONSE 99	**यदि 'नहीं' तो अनुक्रमांक 307 पर जाए If 'no' go to Q 307**
Q 305	जब आपने पहली बार नशीली पदार्थो का इस्तेमाल किया था,तब आप की उम्र क्या थी?<br>How old were you when you first used drugs for recreation<br>**(हम इसमें सिगरेट और तम्बाकू के उपयोग का विचार नहीं कर रहे है)**	---------- उम्र AGE IN COMPLETED YEARS	
Q 306	**<u>पिछले पांच सालों</u>** में क्या आप ने नशीली पदार्थो के कारण हुए तकलीफ़ के लिए इलाज करवाया?<br>Have you received treatment for problem drug use within the LAST FIVE YEARS?<br>**(जैसे बताया गया वही लिखो)**	हाँ YES 1<br>नहीं NO 2<br>जवाब नहीं दिया NO RESPONSE 99	
Q 307	क्या आपने कभी भी सिर्फ मनोरंजन हेतु सुई द्वारा नशीली पदार्थ लिए है क्या?<br>Did you ever take drugs by injection for recreation?<br>**(जैसे बताया गया वही लिखो)**	हाँ YES 1<br>नहीं NO 2<br>पता नहीं DON'T KNOW 88<br>जवाब नहीं दिया NO RESPONSE 99	**यदि 'नहीं' तो अनुक्रमांक 401 पर जाए If 'no' go to Q 401**

पहचान क्रमांक						

गाँव व्यक्ति

Q 308	जब आपने पहली बार सुई द्वारा नशीली पदार्थ लिया था, तब आप की उम्र क्या थी?<br>How old were you when you first took drug by injection for recreation?<br>(जैसे बताया गया वही लिखो)	------------- उम्र AGE IN COMPLETED YEARS<br>पता नहीं DON'T KNOW 88<br>जवाब नहीं दिया NO RESPONSE 99<br>(सही उत्तर का अनुमान लगाए)	
Q 309	ज्यादा तर आप कौनसा नशीला पदार्थ सुई द्वारा लेते है? What drug do you mostly inject?<br>(जैसे बताया गया वही लिखो)	a)----------------<br>b) ----------------<br>c)______________________________	
Q 310	क्या आप सुई द्वारा नशीला पदार्थ लेते समय दवाईयोंका मिश्रण करते है?<br>Do you mix drugs while injecting?<br>(जैसे बताया गया वही लिखो)	हाँ YES 1<br>नहीं NO 2<br>पता नहीं DON'T KNOW 88<br>जवाब नहीं दिया NO RESPONSE 99	
Q 311	दूसरोंके द्वारा इस्तेमाल की हुई पुरानी सुई का इस्तेमाल नशीली पदार्थ लेने के लिए किया है?<br>Have you *ever* used a needle or syringe for taking drugs that had previously been used by someone else?<br>(जैसे बताया गया वही लिखो)	हाँ YES 1<br>नहीं NO 2<br>पता नहीं DON'T KNOW 88<br>जवाब नहीं दिया NO RESPONSE 99	
Q 312	क्या आपने **<u>आखरी बार</u>** नशीली पदार्थ लेते समय , दूसरोंके सुई का इस्तेमाल किया है?<br>During the LAST TIME you injected drug, did you use a needle or syringe that had previously been used by someone else?<br>(जैसे बताया गया वही लिखो)	हाँ YES 1<br>नहीं NO 2<br>पता नहीं DON'T KNOW 88<br>जवाब नहीं दिया NO RESPONSE 99	
Q 313	आपने **<u>आखरी बार</u>** सिर्फ मनोरंजन के लिए कब सुई ली थी (पढ़कर सुनाएं और एक ही जवाब को गोला करे)<br>When was the LAST TIME that you had injected drug for recreation?	आज TODAY 1<br>कल YESTERDAY 2<br>परसों DAY BEFORE YESTERDAY 3<br>पिछले हफ्ते में WITHIN THE LAST ONE WEEK 4<br>पिछले महीने में WITHIN THE LAST ONE MONTH 5<br>पिछले 3 महीने में WITHIN THE LAST THREE MONTHS 6<br>पिछले १साल में WITHIN THE LAST ONE YEAR 7<br>बहुत दिन पहले LONG TIME AGO 8<br>पता नहीं DON'T KNOW 88<br>जवाब नहीं दिया NO RESPONSE 99	

पहचान क्रमांक						

गाँव व्यक्ति

भाग ४. यौन इतिहास–जोड़ीदारों के प्रकार और संख्या

Section 4: Sexual history: numbers and types of partners

मैं आपके शारीरिक संबधो के बारे में कुछ सवाल पूछने जा रहा/रही हूं।कृपया शर्मिंदगी महसूस न करें| मैं आपको फिर से आश्वासन देता/देतीहूं कि जो भी आप कहते हैं वह गोपनीय रहेगा

**अनुक्रमांक** No.	**प्रश्न** Questions and filters	**कोडिंग के प्रकार** Coding categories	**प्रश्न क्रमांक पर जाइये** Skip to
Q 401	क्या आप ने कभी शारीरिक सम्बन्ध बनाया है? Have you *ever* had sexual intercourse? [इस मूल्यांकन का उद्देश्यके लिए, " शारीरिक सम्बन्ध " को योनि या गुदा सेक्स के रूप में परिभाषित किया जाता है।] (Anal/ vaginal sex) **(जैसे बताया गया वही लिखो)**	हाँ YES 1<br>नहीं NO 2<br>जवाब नहीं दिया NO RESPONSE 99	**यदि 'नहीं' तो अनुक्रमांक 501/601 पर जाए** **If 'no' go to Q 501/601**
Q 402	जब आपने **<u>पहली बार</u>** शारीरिक सम्बन्ध बनाया था तब आप की उम्र क्या थी? At what age did you FIRST have sexual intercourse? **(जैसे बताया गया वही लिखो)**	---------- उम्रAGE IN YEARS<br>पता नहीं DON'T KNOW 88<br>जवाब नहीं दिया NO RESPONSE 99	
Q 403	जब आपने **<u>पहली बार</u>** शारीरिक सम्बन्ध बनाया था तब क्या आपने निरोध का इस्तेमाल किया था? Did you use condom while having sex the FIRST time? **(जैसे बताया गया वही लिखो)**	हाँ YES 1<br>नहीं NO 2<br>जवाब नहीं दिया NO RESPONSE 99	
Q 404	आपने **<u>आखरी बार</u>** शारीरिक सम्बन्ध कब किया था? When was the LAST TIME that you had sexual intercourse? **(पढ़कर सुनाएं और एक ही जवाब का चुनाव करे)**	पिछले हफ्ते में WITHIN THE LAST ONE WEEK 1<br>पिछले 3 महीने में WITHIN THE LAST THREE MONTHS 2<br>पिछले १साल में WITHIN THE LAST ONE YEAR 3<br>बहुत दिन पहले LONG TIME AGO 4<br>पता नहीं DON'T KNOW 88<br>जवाब नहीं दिया NO RESPONSE 99	
Q 405	क्या आपने **<u>आखरी बार</u>** शारीरिक सम्बन्ध करते समय निरोध का उपयोग किया था? Did you use condom while having sex the LAST time? **(जैसे बताया गया वही लिखो)**	हाँ YES 1<br>नहीं NO 2<br>जवाब नहीं दिया NO RESPONSE 99	
Q 406	क्या आपके गुप्तांग मे **<u>पिछले एक साल</u>** में कभी घाव हुआ है? Did you have ulcer on your genitalia within the LAST ONE YEAR **(जैसे बताया गया वही लिखो)**	हाँ YES 1<br>नहीं NO 2<br>जवाब नहीं दिया NO RESPONSE 99	

पहचान क्रमांक						

गाँव व्यक्ति

Q 407	क्या आप को कभी भी मलद्वार के रास्ते में **<u>पिछले एक साल</u>** घाव हुआ है ? Did you have ulcer around your anus within the LAST ONE YEAR? **(जैसे बताया गया वही लिखो)**	हाँ YES 1<br>नहीं NO 2<br>जवाब नहीं दिया NO RESPONSE 99	
Q 408	क्या **<u>पिछले एक साल</u>** में आपके गुप्तांग पर छोटी गांठ उत्पन्न हुई है क्या ? Did you have small cauliflower-like growth on your genitalia within the LAST ONE YEAR? **जैसे बताया गया वही लिखो)**	हाँ YES 1<br>नहीं NO 2<br>जवाब नहीं दिया NO RESPONSE 99	
Q 409	क्या **<u>पिछले एक साल</u>** में आपके मलद्वार पर छोटी गांठ उत्पन्न हुई है क्या ? Did you have small cauliflower-like growth around your anus within the LAST ONE YEAR? **(जैसे बताया गया वही लिखो)**	हाँ YES 1<br>नहीं NO 2<br>जवाब नहीं दिया NO RESPONSE 99	
Q 410	क्या **<u>पिछले एक साल</u>** में आपने कभी पेशाब करते समय जलन महसूस की है? Did you have burning sensation during urination within the LAST ONE YEAR? **(जैसे बताया गया वही लिखो)**	हाँ YES 1<br>नहीं NO 2<br>जवाब नहीं दिया NO RESPONSE 99	
Q 411	क्या **<u>पिछले एक साल</u>** में आपके पेशाब में कभी मवाद निकला है? Did you have discharge of pus with urine within the LAST ONE YEAR? **(जैसे बताया गया वही लिखो)**	हाँ YES 1<br>नहीं NO 2<br>जवाब नहीं दिया NO RESPONSE 99	

पहचान क्रमांक						

गाँव व्यक्ति

५ . केवल पुरुषों को पूछने के लिए
Section 5: To be asked to MEN only

अनुक्रमांक No.	प्रश्न Questions and filters	कोडिंग के प्रकार Coding categories	प्रश्न क्रमांक पर जाइये Skip to
Q 501	**विवाहित पुरुषों से पूछिए जिनकी पत्नी जीवित है**<br>**ASK TO MARRIED MEN ONLY WHO HAVE LIVING WIVES**<br>A] क्या आप अपनी पत्नी के साथ शारीरिक सम्बन्धकरते हुए निरोध का प्रयोग करते हैं? Do you use condom while having sex with your wife?<br>यदि "हां" है,<br>B] अपनी पत्नी के साथ शारीरिक सम्बन्ध रखने के दौरान कितनी बार आप निरोध का उपयोग करते हैं?<br>How often do you use condom while having sex with your wife?<br>**(पढ़कर सुनाएं और एक ही जवाब का चुनाव करे)**<br>**[Read out and encircle suitable one**	A] हाँ YES 1<br>नहीं NO 2<br><br>B] हमेशा ALWAYS 1<br>लगभग हमेशा ALMOST ALWAYS 2<br>कभी-कभी SOMETIMES 3<br>शायद ही कभी RARELY 4<br>कभी नहीं NEVER 5	
Q. 502<br><br>Q 502<br><br>Q 502<br><br>Q 502	A] क्या आप नियमित शारीरिक सम्बन्ध बनाने वाली साथी के आलावा किसी अन्य महिला के साथ शारीरिक सम्बन्ध रखते हैं? Do you have sex with your casual partner?<br><br>B] क्या आप नियमित शारीरिक सम्बन्ध बनाने वाली साथी के आलावा किसी अन्य महिला के साथ शारीरिक सम्बन्ध रखते समय निरोध का उपयोग करते हैं? Do you use condom while having sex with your casual partner?<br>C] यदि "हां" है<br>क्या आप नियमित शारीरिक सम्बन्ध बनाने वाली साथी के आलावा किसी अन्य महिला के साथ शारीरिक सम्बन्ध के दौरान कितनी बार आप निरोध का उपयोग करते हैं?<br>How often do you use condom while having sex with your casual partner?<br>**(पढ़कर सुनाएं और एक ही जवाब का चुनाव करे)Read out and encircle suitable one**<br>D) क्या आप नियमित शारीरिक सम्बन्ध बनाने वाली साथी के आलावा किसी अन्य पुरुष के साथ शारीरिक सम्बन्ध रखते हैं?<br>Do you have sex with your casual male partner?	A] हाँ YES 1<br>नहीं NO 2<br><br>B] हाँ YES 1<br>नहीं NO 2<br><br>C] हमेशा ALWAYS 1<br>लगभग हमेशा ALMOST ALWAYS 2<br>कभी-कभी SOMETIMES 3<br>शायद ही कभी RARELY 4<br>कभी नहीं NEVER 5<br><br>D] हाँ YES 1<br>नहीं NO 2	**यदि 'नहीं' तो अनुक्रमांक 502(D) पर जाए**<br>**If 'no' go to Q 502(D)**

पहचान क्रमांक						

गाँव व्यक्ति

Q 502<br><br>Q 502	E] क्या आप नियमित शारीरिक सम्बन्ध बनाने वाली साथी के आलावा किसी अन्य पुरुष के साथ शारीरिक सम्बन्ध रखते समय निरोध का उपयोग करते हैं? Do you use condom while having sex with your casual partner?<br><br>F] यदि "हां" है क्या आप नियमित शारीरिक सम्बन्ध बनाने वाली साथी के आलावा किसी अन्य पुरुष के साथ शारीरिक सम्बन्ध रखते के दौरान कितनी बार आप निरोध का उपयोग करते हैं?<br>How often do you use condom while having sex with your casual partner?<br>**(पढ़कर सुनाएं और एक ही जवाब का चुनाव करे)Read out and encircle suitable one**	E] हाँ YES 1<br>नहीं NO 2<br><br>F] हमेशा ALWAYS 1<br>लगभग हमेशा ALMOST ALWAYS 2<br>कभी-कभी SOMETIMES 3<br>शायद ही कभी RARELY 4<br>कभी नहीं NEVER 5	
Q 503	A] क्या आप देह व्यापर करने वाले महिला के साथ करनेवाले महिलाके साथ शारीरिक सम्बन्ध रखते हैं?<br>Do you have sex with female sex worker?<br><br>B] देह व्यापर करने वाले महिला के साथ करने वाले महिला के साथ शारीरिक सम्बन्धके दौरान क्या आप निरोध का उपयोग करते हैं?<br>Do you use condom while having sex with female sex worker?<br><br>C] यदि "हां" है,<br>देह व्यापर करने वाले महिला के साथ करनेवाले महिला के साथ शारीरिक सम्बन्ध के दौरान आप कितनी बार निरोध का उपयोग करते हैं?<br>How often do you use condom while having sex with your casual partner?<br>**(पढ़कर सुनाएं और एक ही जवाब का चुनाव करे)] Read out and encircle suitable one.**	A] हाँ YES 1<br>नहीं NO 2<br><br>B] हाँ YES 1<br>नहीं NO 2<br><br>C] हमेशा ALWAYS 1<br>लगभग हमेशा ALMOST ALWAYS 2<br>कभी-कभी SOMETIMES 3<br>शायद ही कभी RARELY 4<br>कभी नहीं NEVER 5	
Q 504	क्या आपको **पिछले एक साल** में अण्डकोष में दर्द भरी सूजन हुई है?<br>Did you have painful scrotal swelling within the last ONE YEAR?**(जैसे बताया गया वही लिखो)**	हाँ YES 1<br>नहीं NO 2<br>पता नहीं DON'T KNOW 88<br>जवाब नहीं दिया NO RESPONSE 99	

पहचान क्रमांक						

गाँव व्यक्ति

Q 505	क्या आपने कभी पुरुष साथी के साथ शारीरिक संबंध किया है?<br>Have you ever had sex with a male partner?<br>**(जैसे बताया गया वही लिखो)**	हाँ YES 1<br>नहीं NO 2<br>पता नहीं DON'T KNOW 88<br>जवाब नहीं दिया NO RESPONSE 99	**यदि 'नहीं' तो अनुक्रमांक 507 पर जाए If 'no' go to Q 507**
Q 506	<u>**आखिरी बार**</u> जब आप पुरुष साथी के साथ शारीरिक संबंध रखते थे, तो क्या आपने निरोध का इस्तेमाल किया था?<br>The LAST TIME you had sex with a male partner, did you use condom?<br>**(पढ़कर सुनाएं और एक ही जवाब का चुनाव करे)Read out and encircle suitable one**	हमेशा ALWAYS 1<br>ज्यादातर MOST OF THE TIMES 2<br>कभी कभी SOMETIMES 3<br>कभी नहीं NEVER 4	
Q 507	क्या आपने कभी पैसे या उपहार के बदले यौन संबंध किया है?<br>Did you ever have sex in exchange of money or gift?<br>**(जैसे बताया गया वही लिखो)**	हाँ YES 1<br>नहीं NO 2<br>पता नहीं DON'T KNOW 88<br>जवाब नहीं दिया NO RESPONSE 99	**यदि 'नहीं' तो विभाग 7 पर जाए If 'no' go to Section 7**
Q 508	पैसे या उपहार के बदले शारीरिक सम्बन्ध करते समय क्या आप निरोध का उपयोग करते हैं?<br>Do you use condoms while having sex in exchange of money or gift?<br>**(जैसे बताया गया वही लिखो)**	हाँ YES 1<br>नहीं NO 2<br>पता नहीं DON'T KNOW 88<br>जवाब नहीं दिया NO RESPONSE 99	
Q 509	पैसे या उपहार के बदले शारीरिक संबंध करते समय निरोध का कितनी बार उपयोग करते हैं?<br>How frequently do use condoms while having sex in exchange of money or gift?<br>**(पढ़कर सुनाएं और एक ही जवाब का चुनाव करे)Read out and encirclesuitable one**	हमेशा ALWAYS 1<br>ज्यादातर MOST OF THE TIMES 2<br>कभी कभी SOMETIMES 3<br>कभी नहीं NEVER 4	

पहचान क्रमांक						

गाँव   व्यक्ति

भाग ६ . केवल महिला को पूछने के लिए
Section 6: To be asked to WOMEN only

अनुक्रमांक No.	प्रश्न Questions and filters	कोडिंग के प्रकार Coding categories	प्रश्न क्रमांक पर जाइये Skip to
Q. 601	क्या **पिछले एक साल** में कभी आप के योनी से सफ़ेद स्राव/ पानी आ रहा था ?<br>Did you have white vaginal discharge within the LAST ONE YEAR?<br>**(जैसे बताया गया वही लिखो)**	हाँ YES 1<br>नहीं NO 2<br>जवाब नहीं दिया NO RESPONSE 99	
Q. 602	क्या **पिछले एक साल** में शारीरिक सम्बन्ध करते समय आप के पेट के निछले हिस्से में दर्द हुआ था?<br>Did you have lower abdominal pain during sexual intercourse within the LAST ONE YEAR?<br>**(जैसे बताया गया वही लिखो)**	हाँ YES 1<br>नहीं NO 2<br>जवाब नहीं दिया NO RESPONSE 99	
Q. 603	क्या आपका कोई नियमित शारीरिक सम्बन्ध बनाने वाला साथी है?<br>(विवाहित महिला के लिए उसका पति)<br>Do you have a regular sex partner (husband in case of married woman)?<br>**(जैसे बताया गया वही लिखो)**	हाँ YES 1<br>नहीं NO 2<br>जवाब नहीं दिया NO RESPONSE 99	**यदि 'नहीं' तो अनुक्रमांक 608 पर जाए**<br>**If "no "go to Q 608**
Q .604	क्या आपका नियमित शारीरिक सम्बन्ध बनाने वाला साथी शराब पीता है?<br>Does your regular sex partner use alcohol?<br>**(जैसे बताया गया वही लिखो)**	हाँ YES 1<br>नहीं NO 2<br>पता नहीं DON'T KNOW 88<br>जवाब नहीं दिया NO RESPONSE 99	
Q. 605	क्या आपका नियमित शारीरिक सम्बन्ध बनाने वालासाथी नशीली दवा का प्रयोग करता है?<br>(निकोटिन और तम्बाकू छोड़ के और कुछ लेता)<br>Does your regular sex partner use drugs?<br>**(Nicotine and Tobacco NOT included)**	हाँ YES 1<br>नहीं NO 2<br>पता नहीं DON'T KNOW 88<br>जवाब नहीं दिया NO RESPONSE 99	**यदि 'नहीं 'तो अनुक्रमांक 608 पर जाए**<br>**If "no "go to Q 608**
Q. 606	क्या आपका नियमित शारीरिक सम्बन्ध बनाने वाला साथी शराब के नशे में आप को गाली देता है?<br>Does your regular sex partner abuse you verbally (calling you names or saying things that hurt you) under the influence of drugs / alcohol?<br>**(जैसे बताया गया वही लिखो)**	हाँ YES 1<br>नहीं NO 2<br>पता नहीं DON'T KNOW 88<br>जवाब नहीं दिया NO RESPONSE 99	
Q. 607	क्या आपका नियमितशारीरिक सम्बन्ध बनाने वालासाथी शराब के नशे में आपको शारीरिक हानि (धक्का देता हैं या मार पिट)पहुचाता है?<br>Does your regular sex partner abuse you physically (push, hit or hurt you physically) under the influence of drugs / alcohol?	हाँ YES 1<br>नहीं NO 2<br>जवाब नहीं दिया NO RESPONSE 99	

पहचान क्रमांक						

गाँव व्यक्ति

Q .608	A) क्या आप अन्य पुरुष साथी के साथ शारीरिक सम्बन्ध (अपने पति / प्रेमी के अलावा अन्य लैंगिक साथी) बनाते है?<br>Do you have a casual sex partner?<br><br>B] क्या आप अपने किसी अन्य पुरुष साथी के साथ शारीरिक सम्बन्ध के दौरान निरोध का उपयोग करते हैं (अपने पति / प्रेमी के अलावा अन्य लैंगिक साथी)<br>Does your casual partner (sex partner other than your husband/boyfriend) use condom while having sex with you?<br><br>**C]** यदि 608(A) को 'हा' है<br>अपने किसी अन्य पुरुष साथी के साथ शारीरिक संबंध के दौरान आप कितनी बार निरोध का उपयोग करते हैं?<br>How often does your casual partner use condom while having sex with you?<br>**(जैसे बताया गया वही लिखो)**	A] हाँ YES 1<br>नहीं No 2<br><br>B] हाँ YES 1<br>नहीं No 2<br><br>C] हमेशा ALWAYS1<br>ज्यादातर MOST OF THE TIMES 2<br>कभी कभी SOMETIMES 3<br>कभी नहीं NEVER 4	**यदि 'नहीं 'तो अनुक्रमांक 701 पर जाए**<br>**If "no "go to Q 701**

Section 7: Thanking and offer for blood test

भाग ७: रक्त परीक्षण के लिए धन्यवाद और प्रस्ताव

I thank you for your responses.As part of this ASSESSMENT we are offering free voluntary confidential blood test facility

मैं आपकी प्रतिक्रियाओं के लिए धन्यवाद करता/करती हु। इस आकलन के हिस्से के रूप में हम मुफ्त स्वैच्छिक गोपनीय रक्त परीक्षण सुविधा की पेशकश कर रहे हैं।

No.	Questions and filters	Coding categories	Skip to
Q.701	Are you interested in accepting this offer?<br>आप खून की जांचे करना चाहते हो?<br><br>**(जैसे बताया गया वही लिखो)**	हाँ YES 1<br>नहीं NO 2<br>जवाब नहीं दिया NO RESPONSE 99	**यदि 'नहीं 'तो विभाग 8 पर जाए**<br>**If "no "go to section 8**
Q.702	Please let us know if you are<br>कृपया हमें बताईये<br><br>**(जैसे बताया गया वही लिखो)**	जाँच करना चाहते है और उनके परिणाम जानना चाहते है I WILLING TO TAKE THE TEST AND LIKE TO HAVE THE TEST RESULTS 1<br>जाँच करना चाहते है पर उनके परिणाम नहीं जानना चाहते WILLNG TO TAKE THE TEST AND DO NOT WANT THE TEST RESULTS 2	

If respondent, wishes to undergo the tests, please arrange to draw blood immediately after completion of interview at the designated site following aseptic procedure. Ensure that the interviewee's code written on this questionnaire is marked also on the test tube containing the blood sample collected from the respondent.

If the respondent wants to have the test results, please issue a card (Link card) with the interviewee's code and other personal identifiers written on it, with which the respondent will be able to access counseling, test result report and referral services as necessary.

The following section will be filled in by data entry operators on the hard copy as well as soft copy after receiving the results against each ID code from the respective counselors.

पहचान क्रमांक						

गाँव व्यक्ति

Q.703	Was the specimen of blood taken from the willing respondent?	YES 1<br>NO 2	
Q.704	Test for HIV antibody	POSITIVE 1<br>NEGATIVE 2<br>SAMPLE NOT ADEQUATE 3<br>SAMPLE LOST/ TEST TUBE BROKEN 4	
Q.705	Test for RPR(test for syphilis)	POSITIVE 1<br>NEGATIVE 2<br>SAMPLE NOT ADEQUATE 3<br>SAMPLE LOST/ TEST TUBE BROKEN 4	
Q.706	Test for TPHA (test for syphilis)	POSITIVE 1<br>NEGATIVE 2<br>SAMPLE NOT ADEQUATE 3<br>SAMPLE LOST/ TEST TUBE BROKEN 4	
Q.707	Test for Hepatitis surface Antigen (HBsAg)	POSITIVE 1<br>NEGATIVE 2<br>SAMPLE NOT ADEQUATE 3<br>SAMPLE LOST/ TEST TUBE BROKEN 4	
Q.708	Test for Hepatitis C antibody	POSITIVE 1<br>NEGATIVE 2<br>SAMPLE NOT ADEQUATE 3<br>SAMPLE LOST/ TEST TUBE BROKEN 4	
Q.709	Test for Hepatitis C RNA	POSITIVE 1<br>NEGATIVE 2<br>SAMPLE NOT ADEQUATE 3<br>SAMPLE LOST/ TEST TUBE BROKEN 4	

Section 8: Current Treatment taking history

भाग ८: वर्तमान उपचार इतिहास

I thank you for your participation in the study. As part of this ASSESSMENT I would like to know about treatments you are currently receiving

मैं अध्ययन में आपकी भागीदारी के लिए धन्यवाद। इस आकलन के हिस्से के रूप में मैं उन उपचारों के बारे में जानना चाहता /चाहती हूं जिन्हें आप इस समय में प्राप्त कर रहे हैं।

No.	Questions and filters	Coding categories	Skip to
Q.801	Are you currently taking treatment for some illnesses?<br>क्या आप इस समय में कुछ बीमारियों के लिए इलाज कर रहे हैं?	हाँ YES 1<br>नहीं NO 2<br>जवाब नहीं दिया NO RESPONSE 99	
Q.802	Please tell me what are those diseases<br>कृपया मुझे बताएं कि ये बीमारियां क्या हैं?	a.……………………………….रोग का नाम<br>(Name of the disease)<br>b.…………………………… कोई अन्य बीमारी	

पहचान क्रमांक						

गाँव व्यक्ति

	(जैसा कि कहा गया वैसे लिखे)	(Any other disease)<br>c……………………………. कोई अन्य बीमारी<br>(Any other disease)<br>d……………………………. कोई अन्य बीमारी	
Q.803	For disease a…………… since when are you taking medicines<br>बीमारी' ए' के लिए कब से आप दवा ले रहे हैं?	……………………………………………<br>(PLEASE RECORD THE RESPONSE AS NARRATED)<br>**(जैसा कि कहा गया वैसे लिखे)**	
Q.804	For disease a…………… where from do you get the medicines<br>बीमारी 'ए' के लिए कहाँ से आप दवाएं प्राप्त करते हैं?	……………………………………………<br>(PLEASE RECORD THE RESPONSE AS NARRATED)<br>**(जैसा कि कहा गया वैसे लिखे)**	
Q.805	For disease b…………… since when are you taking medicines<br>बीमारी 'बी' के लिए कब से आप दवा ले रहे हैं?	……………………………………………<br>(PLEASE RECORD THE RESPONSE AS NARRATED)<br>**(जैसा कि कहा गया वैसे लिखे)**	
Q.806	For disease b…………… where from do you get the medicines<br>बीमारी 'बी' के लिए कहाँ से आप दवाएं प्राप्त करते हैं?	……………………………………………<br>(PLEASE RECORD THE RESPONSE AS NARRATED)<br>**(जैसा कि कहा गया वैसे लिखे)**	
Q.807	For disease c…………… since when are you taking medicines<br>बीमारी 'सी' के लिए कब से आप दवा ले रहे हैं?	……………………………………………<br>(PLEASE RECORD THE RESPONSE AS NARRATED)<br>**(जैसा कि कहा गया वैसे लिखे)**	
Q.808	For disease c…………… where from do you get the medicines<br>बीमारी 'सी' के लिए कहाँ से आप दवाएं प्राप्त करते हैं?	……………………………………………<br>(PLEASE RECORD THE RESPONSE AS NARRATED)<br>**(जैसा कि कहा गया वैसे लिखे)**	
Q.809	For disease d…………… since when are you taking medicines<br>बीमारी 'डी' के लिए कब से आप दवा ले रहे हैं?	……………………………………………<br>(PLEASE RECORD THE RESPONSE AS NARRATED)<br>**(जैसा कि कहा गया वैसे लिखे)**	
Q.810	For disease d…………… where from do you get the medicines<br>बीमारी 'डी' के लिए कहाँ से आप दवाएं प्राप्त करते हैं?	……………………………………………<br>(PLEASE RECORD THE RESPONSE AS NARRATED)<br>**(जैसा कि कहा गया वैसे लिखे)**	
