# Supplementary material for: Unsterile injection equipment associated with HIV outbreak and an extremely high prevalence of HCV—A case-control investigation from Unnao, India
Source: PLoS One. 2020 Dec 4;15(12):e0243534. doi: 10.1371/journal.pone.0243534 (PMC7717531; doi:10.1371/journal.pone.0243534)
Supplement: Attachment — Submitted filename: Response to Reviewers.docx [file pone.0243534.s002.docx]

“RESPONSE TO EDITOR AND REVIEWERS”

EDITOR’S COMMENTS

1. Please ensure that your manuscript meets PLOS ONE's style requirements, including those for file naming. The PLOS ONE style templates can be found at

<https://journals.plos.org/plosone/s/file?id=wjVg/PLOSOne_formatting_sample_main_body.pdf> and

<https://journals.plos.org/plosone/s/file?id=ba62/PLOSOne_formatting_sample_title_authors_affiliations.pdf>

We have ensured that manuscript meets PLOS ONE's style requirements, including those for file naming.

2. Please include additional information regarding the survey or questionnaire used in the study and ensure that you have provided sufficient details that others could replicate the analyses. If you developed and/or translated a questionnaire as part of this study and it is not under a copyright more restrictive than CC-BY, please include a copy, in both the original language and English, as Supporting Information.

We have provided a copy of questionnaire developed by us for the study.

3. PLOS ONE requires that experiments, statistics, and other analyses are performed to a high technical standard and are described in sufficient detail to allow another researcher to reproduce the experiments (<https://journals.plos.org/plosone/s/criteria-for-publication#loc-3>), and that the conclusions presented in the manuscript are supported by the data (<https://journals.plos.org/plosone/s/criteria-for-publication#loc-4>). Please therefore remove the statements throughout your manuscript that HSV-2 IgG is an objective biomarker for sexual lifestyle or exposure, since HSV-2 transmission can occur by other modes, including vertical transmission from mother to child.

We have removed the statements throughout the manuscript- “HSV-2 IgG is an objective biomarker for sexual lifestyle or exposure”

 4.We note that you have indicated that data from this study are available upon request. PLOS only allows data to be available upon request if there are legal or ethical restrictions on sharing data publicly. For information on unacceptable data access restrictions, please see <http://journals.plos.org/plosone/s/data-availability#loc-unacceptable-data-access-restrictions>.

### Data contain potentially identifying or sensitive patient information / study location specific information which, if disclosed, may lead to stigma and discrimination. Due to ethical restrictions, data can be made available on request to Institutional Ethics committee of ICMR-NARI, Pune. (Email id- [ecnari@nariindia.org](mailto:ecnari@nariindia.org)) as well as Project Director, Uttar Pradesh State AIDS Control Society (UPSACS). (Email id- pd.upsacs@gmail.com).

5. PLOS requires an ORCID iD for the corresponding author in Editorial Manager on papers submitted after December 6th, 2016. Please ensure that you have an ORCID iD and that it is validated in Editorial Manager. To do this, go to ‘Update my Information’ (in the upper left-hand corner of the main menu), and click on the Fetch/Validate link next to the ORCID field. This will take you to the ORCID site and allow you to create a new iD or authenticate a pre-existing iD in Editorial Manager. Please see the following video for instructions on linking an ORCID iD to your Editorial Manager account: <https://www.youtube.com/watch?v=_xcclfuvtxQ>

 ORCID ID has been updated

6.We note that [Figure(s) 1] in your submission contain [map/satellite] images which may be copyrighted. All PLOS content is published under the Creative Commons Attribution License (CC BY 4.0), which means that the manuscript, images, and Supporting Information files will be freely available online, and any third party is permitted to access, download, copy, distribute, and use these materials in any way, even commercially, with proper attribution. For these reasons, we cannot publish previously copyrighted maps or satellite images created using proprietary data, such as Google software (Google Maps, Street View, and Earth). For more information, see our copyright guidelines: <http://journals.plos.org/plosone/s/licenses-and-copyright>.

We have excluded map (Fig 1 from earlier version) from revised manuscript and content of the map has been tabulated.

Additional Editor Comments:

I agree with the two reviewers that the paper has interest and presents on an important topic. However, in its present form it does't meet criteria for publishing. Re-organization and focus will significantly improve the manuscript. In addition to editorial (grammar) review, this paper could be shortened by 20% by focusing on the main findings.

We have used Grammarly tool for grammar review. We have also reorganized and reduced our content by focusing on main findings to improve manuscript.

**Reviewer 1**

1. The article has many typographical errors and the authors should employ the use of GRAMMARLY, (it is a free tool) to help in edits throughout the document.

We have used Grammerly tool which was immensely helpful for grammar review.

2. The methodology needs to be re-written. Study area. Study design, Study population. Sample size determination. Sampling technique. Dependent and independent variables. eligibility criteria. Data collection tool and procedure; was this interviewer assisted or administered? remember to include who took the blood samples and how? Where was the tool adapted from? was it validated? any pretests? Data analysis: SPSS? Descriptive statistics computed? Ethical considerations: ethics committee and how consent was obtained from participants, how research assistants were trained on e.g confidentiality. Keeping respondents identities anonymous?

Reporting them under these sub sections, helps for clarity. many of these sub themes were either totally absent or mixed up together.

We have reorganized our manuscripts and incorporated all aforementioned suggestions in methodology section.

3. Discussion. Why is the first line mentioning secondary data analysis? Please clarify.

We shifted it to last part of the first paragraph of discussion.

4. The second paragraph that mentions bias should be moved to the last paragraph and how these were addressed should be included.

We have moved bias part to the last paragraph

5. There was no conclusion or recommendation. Please kindly include this

We have included conclusion section in the manuscript.

6. An algorithm for how this cases and controls were selected should be done.

We have added an algorithm (Fig 1) pertaining to selection of cases and controls.

7. The conclusion and recommendations should please be in response to the objectives of the paper, and the results respectively.

Conclusion in response to the objectives of the paper and results have been included.

8. This article from pubmed can be a great guide in re-organizing the article.

https://www.ncbi.nlm.nih.gov/pmc/articles/PMC7049326/

Determinants of Anemia among HIV-Positive Children on Highly Active Antiretroviral Therapy Attending Hospitals of North Wollo Zone, Amhara Region, Ethiopia, 2019: A Case-Control Study

We have followed the structure as depicted in aforementioned article suggested by reviewer.

**Reviewer 2**

1. The population is not adequately described ,especially for an international reader. A small table including the total population of Uttar Pradesh, Unnao district, Bangarmau block and Premganj township-villages of Karimuddinpur and Chakmeerapur should be included.

We have adequately described population in revised manuscript with reference to

Census 2011.

2. The history of blood transfusion and invasive surgical procedures is not included in the tables.

We have added aforementioned history in table 4.

3. The statistics in the multivariate analysis are not adequate. I recommend to present models including "Received intramuscular injection in last five years" or "Received intravenous fluid for treatment in last five years" in addition to the current model including "Syringe & needle used during intramuscular injection in last five years". The last model may be problematic since the potencial for recall bias is high.

We added “receipt of intramuscular injection” and rerun multivariate regression analysis and results have been produced in table 5.

4. In figure 3 the legend does not describe the meaning of different colours.

We have excluded fig 3 and new phylogenetic tree has been added. We also modified methodology pertaining to phylogenetic analysis

5. I recommend modification of the title to emphasise the main findings of the study e.g. unsterilized injection equipment for medical use is a cause of the HIV outbreak and extremely high prevalence of HCV.

As per suggestion, we have modified the title of the manuscript.

6. The authors should discuss the generalizability of the HCV findings for Unnao district and Uttar Pradesh. Their discussion is very confusing in this very important issue.

We have discussed generalizability of the HCV findings for Unnao district and Uttar

Pradesh. We have simplified the discussion and made it more lucid.
